# Remotely constraining the temporal evolution of offshore oil systems

**DOI:** 10.1038/s41598-018-37884-x

**Published:** 2019-02-04

**Authors:** Alexander J. Corrick, David Selby, David M. McKirdy, Philip A. Hall, Se Gong, Christine Trefry, Andrew S. Ross

**Affiliations:** 1 Department of Earth Sciences, School of Physical Sciences, University of Adelaide, Adelaide, SA 5005 Australia; 2 Department of Earth Sciences, Durham University, Durham, DH1 3LE UK; 3 State Key Laboratory of Geological Processes and Mineral Resources, School of Earth Resources, China University of Geosciences, Wuhan, 430074 Hubei China; 4 Energy, CSIRO, North Ryde, NSW 2113 Australia; 5 Energy, CSIRO, Kensington, WA 6151 Australia

## Abstract

An understanding of the temporal evolution of a petroleum system is fundamental to interpreting where hydrocarbons may be trapped in the subsurface. However, traditional exploration methods provide few absolute constraints on the timing of petroleum generation. Here we show that ^187^Re/^187^Os geochronology may be applied to natural crude oil seepage to determine when petroleum generation occurred in offshore sedimentary basins. Using asphaltites collected from the South Australian coastline, our determined Re-Os age (68 ± 15 million years ago) is consistent with their derivation from a Late Cretaceous source rock in the nearby Bight Basin, an interpretation similarly favoured by source-specific biomarker constraints. Furthermore, the calculated initial ^187^Os/^188^Os composition of the asphaltites, a value inherited from the source rock at the time of oil generation, suggests that the source rock represents the later stage of Oceanic Anoxic Event 2. Our results demonstrate a new approach to identifying the origin of crude oils encountered in coastal environments by providing direct constraints on the timing of petroleum generation and potential source rock intervals in poorly characterised offshore sedimentary basins prior to exploratory drilling.

## Introduction

Natural seepage is a major contributor of oil to the marine environment, estimated at ~600,000 tonnes per year^1^. When entrained in prevailing ocean currents, this seepage may strand along the coastline as liquid oil, viscous tar or semi-solid bitumen^2–4^. The distance crude oils are transported by ocean currents may be short if the seepage site is near the coastline^5^. However, there is evidence of significantly longer transport, where bitumens are found on distant foreign coastlines^3^. The geochemistry of these crude oils can be used to characterise and identify their respective parent petroleum systems and potentially distinguish them from anthropogenic inputs of oil such as tanker spillage^6^.

The traditional approach to geochemically characterising and correlating crude oils utilises carbon isotopes and biomarkers to ascertain the depositional environment and organic facies of their source rocks^7^. Although specific biomarkers may broadly constrain the depositional age of a source rock and provide an estimate of its thermal maturity, none of these analytical tools yields an absolute age. Understanding the timing of oil generation from the source rock is vital to assessing the potential migration pathways and structural traps which form viable hydrocarbon reservoirs. Consequently, petroleum exploration relies heavily on a theoretical timing of hydrocarbon generation determined from basin modelling^8^. These models are generally based on assumptions about the source rocks that may be active in the basin, which in turn introduces a component of uncertainty that may affect exploration prospects.

A major recent advance in petroleum geochemistry has been the ability to directly determine the timing of hydrocarbon generation from the source rock using the decay of ^187^Re to ^187^Os within the produced oil^9–14^. Studies using naturally formed oils and those produced via artificial maturation of source rocks in a laboratory setting have also demonstrated that the initial ^187^Os/^188^Os composition of an oil (Os_i_) is inherited directly from the source rock at the time of generation^13–15^. Therefore, comparison of this Os_i_ value of an oil to the ^187^Os/^188^Os composition of a proposed source rock at the time of generation (Os_g_) may be used to aid oil-to-source correlations, should data on the source rock be available^11,14,16^. However, previous applications of Re-Os dating using oils and bitumens have been restricted to samples collected *in-situ*, rather than examples released from the subsurface into the marine environment through natural seepage. The successful application of Re-Os geochronology to seep emissions will permit the timing of oil generation to be constrained prior to exploratory drilling.

**Figure 1:**
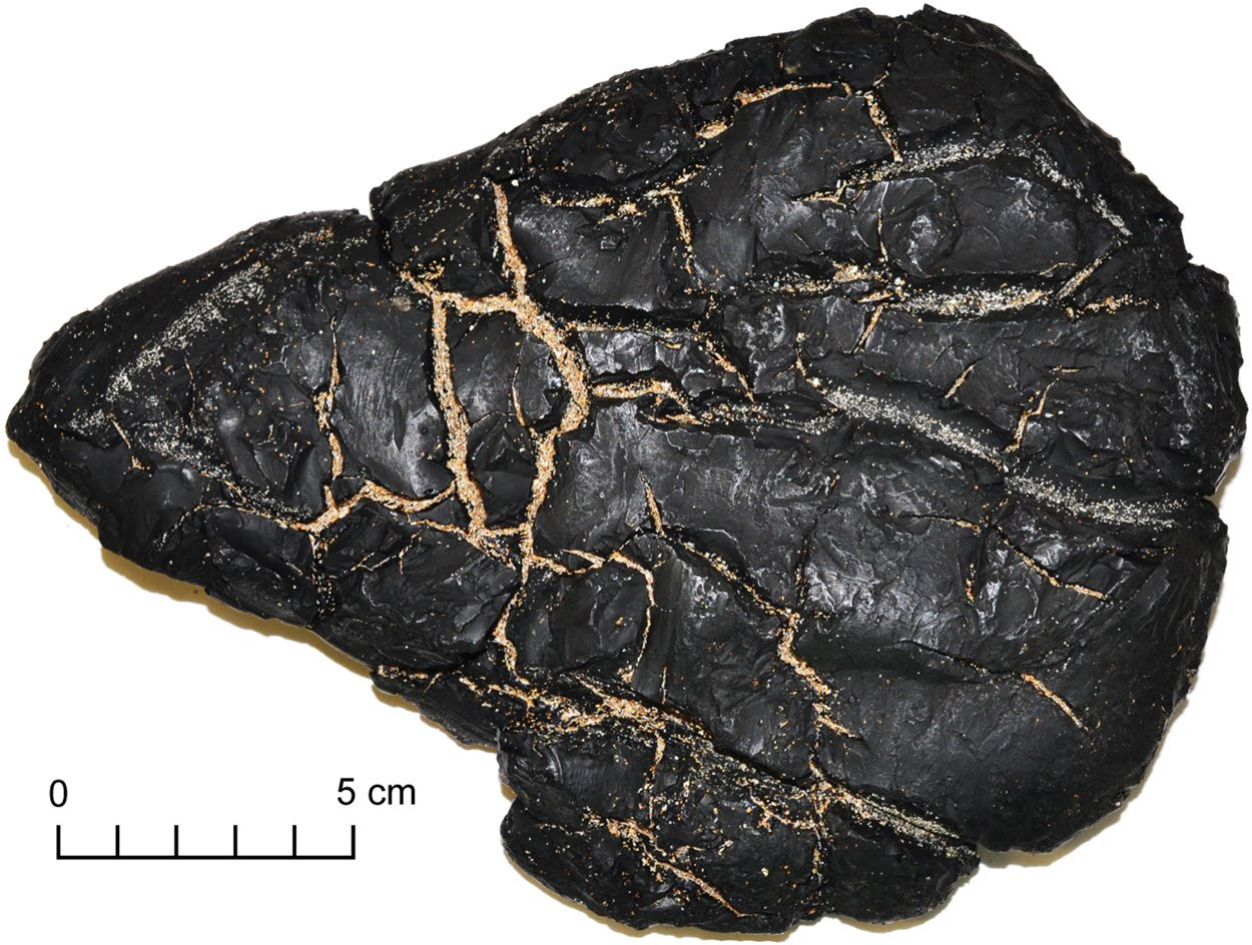
Large asphaltite exhibiting deep shrinkage cracks following devolatilization of low-molecular-weight hydrocarbons. Sample W13/007507, collected from Waitpinga Beach, South Australia in 2014.

Australia’s southern margin collects bitumens emitted from a variety of different petroleum systems^2,3,17,18^. One of the rarest, but most distinctive, types encountered is known as asphaltite^2,17^. These bitumens are easily identified by their striking appearance defined by deep shrinkage cracks, conchoidal fracture and jet-black colour (Fig. 1). A combination of highly reproducible source-specific biomarkers and other hydrocarbons constrains the origin of these asphaltites to a single oil family expelled early in the oil generation window from a Mesozoic marine shale deposited under anoxic/sulfidic conditions^2,17^. Their biomarker composition is unique, and yet to be linked to any produced oil worldwide^19^. This suggests that the asphaltites are not the result of anthropogenic spillage, but rather the product of seafloor seepage from an unidentified petroleum system. Despite their prior extended immersion in seawater, recently stranded asphaltite specimens also preserve high abundances of slightly water-soluble aromatic hydrocarbons such as methylnaphthalenes and phenanthrene, indicating that the parent petroleum system is proximal to their region of stranding^2,17^. Presently, the origin of these asphaltite strandings is attributed to a source rock deposited during the Late Cretaceous global oceanic anoxic event (OAE2) at the Cenomanian-Turonian boundary (93.9 Ma) within the nearby Bight Basin^17,20^. This predominantly offshore Jurassic-Cretaceous rift basin formed during the separation of Australia and Antarctica^21,22^ and remains relatively unexplored. However, it is proposed to contain marine-derived source rocks deposited during the latest Cenomanian to earliest Turonian^20^.

**Figure 2:**
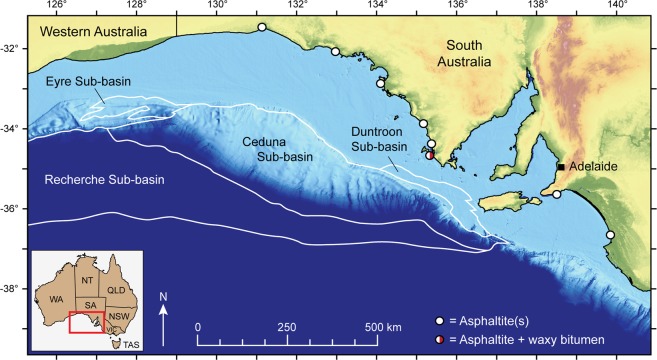
Stranding locations of analysed samples and the offshore Jurassic-Cretaceous sub-basins of the Bight Basin. See Table S1 for further sample collection information.

Here we present Re-Os data from asphaltites collected from the South Australian coastline (n = 11; Fig. 2; Table S1) during annual beach surveys in 2014–2016, as part of the Great Australian Bight Research Program^18,23^. We show that the timing of oil generation in an offshore sedimentary basin may be constrained directly using hydrocarbon seepage. This information may then be used to inform, refine or support petroleum systems models for assessing offshore exploration prospects. We then use the calculated asphaltite Os_i_ value to assess if their origin is consistent with an OAE2 source rock. Our findings demonstrate that the Re-Os isotope systematics of hydrocarbon seepage can provide important information constraining the evolution of offshore oil systems prior to costly exploratory drilling.

## Results

### Asphaltite Re-Os data

**Figure 3:**
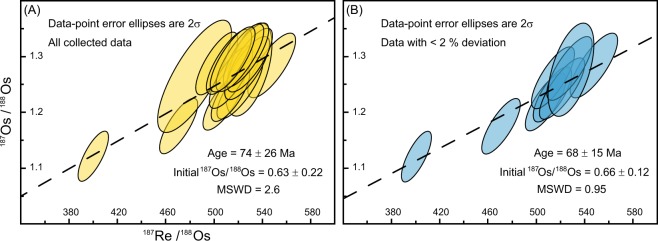
Re-Os isochron diagrams for South Australian asphaltites. (**A**) Regression of all Re-Os data (n = 16) from asphaltite interiors, weathered exteriors and replicate analyses yields an age of 74 ± 26 Ma. (**B**) Regression of asphaltite data with <2% deviation from the regression of all data points (n = 9; Table S3) yields an age of 68 ± 15 Ma.

The asphaltites contain between 2.59 and 4.02 parts per billion (ppb) Re and 24.6 to 43.1 parts per trillion (ppt) Os. The ^187^Re/^188^Os ratios are high (400.5–547.8) and positively correlate with the ^187^Os/^188^Os ratios (1.122–1.303). Regression of all the asphaltite Re-Os data from sample interiors, exteriors and replicate analyses (n = 16; Table S2) yields a Model 3 age (for which the scatter in the data exceeds that expected from analytical precision)^24^ of 74 ± 26 Ma, with an Os_i_ of 0.63 ± 0.22 and a degree of fit (mean square of weighted deviates; MSWD) value of 2.6 (Fig. 3A). The low precision of this age (35%) can be attributed to specific data points which deviate from the best-fit regression line (i.e. the isochron). A refined Re-Os age may therefore be determined by limiting the regression to the data points with low deviation from the predicted ^187^Os/^188^Os value determined from the entire dataset^25^. A revised regression restricted to samples with less than 2% deviation from the regression line (n = 9; Table S3) yields a Model 1 age (for which the scatter in data is consistent with the uncertainties attributable to analytical precision)^24^ of 68 ± 15 Ma, an Os_i_ of 0.66 ± 0.12 and a MSWD of 0.95 (Fig. 3B). Of the seven data points excluded, five were replicate analyses of counterparts that deviated <2% from the initial regression and were still included. The remaining two excluded data points were obtained from specimens with no replicate analyses but returned ^187^Os/^188^Os values comparable to the other excluded data points. Samples with ^187^Os/^188^Os values that differ from the majority of the dataset could be interpreted as having originated from a second source rock. However, this is not the case here because the biomarker geochemistry of all samples analysed is highly consistent (Table S4).

### Extent of alteration in the marine environment

**Figure 4:**
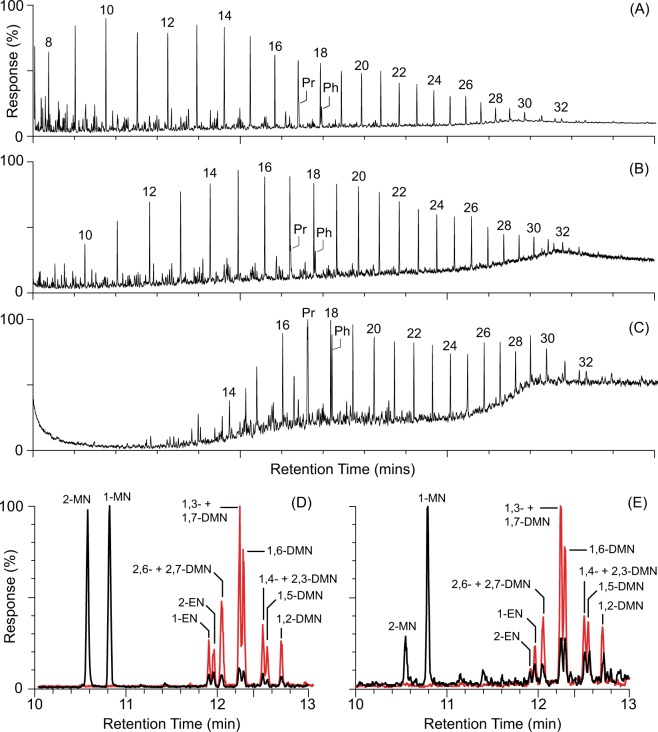
GC-MS chromatograms illustrating the variation in degradation across the suite of analysed asphaltites. (**A**) Whole-oil total ion chromatogram (TIC) of a minimally degraded asphaltite (W13/007976). (**B**) Whole-oil TIC of a moderately degraded asphaltite (W13/007507) showing depletion of low-molecular-weight *n*-alkanes. (**C**) Whole-oil TIC of a highly degraded asphaltite (W13/007668) showing loss of gasoline and kerosene-range alkanes. (**D**) Selected ion monitoring (SIM) chromatograms (black = *m/z* 142, red = *m/z* 156) of the aromatic fraction of a fresh asphaltite (W13/007976), preserving abundant methylnaphthalenes, ethylnaphthalenes and dimethylnaphthalenes. (**E**) SIM chromatograms of the aromatic fraction of a highly degraded asphaltite (W13/007668), displaying prominent loss of 2-MN, 2-EN and 1,5-DMN due to weathering. Key: peak numbers in whole-oil TIC chromatograms correspond to *n*-alkane carbon chain length; Pr = pristane; Ph = phytane; MN = methylnaphthalene; EN = ethylnaphthalene; DMN = dimethylnaphthalene. Geochemical data and assigned level of degradation for each sample is listed in Table S4. The criteria used to define each level of alteration are listed in Table S5.

The majority of the Re and Os in a crude oil is located in its asphaltene fraction, which is highly resistant to the effects of biodegradation^16^. However, to test if exposure to seawater influenced the Re-Os systematics of the asphaltites, the extent of degradation (Fig. 4) in each specimen was assessed based on the alteration and removal of key compounds by evaporation, water washing and biodegradation (Table S5). This assessment revealed that variation in ^187^Re/^188^Os and ^187^Os/^188^Os ratios in the sample suite is not systematic with increasing alteration. Furthermore, comparison of the weathered exteriors (top 2 mm) of four asphaltites with their respective interiors all returned ratios within the range of analytical uncertainty (Table S2). Finally, the Re-Os characteristics of the asphaltites were compared to those of a waxy bitumen specimen (W13/007697) recovered from the same coastline and known to originate from one of several distant Cenozoic lacustrine petroleum systems in the Indonesian archipelago^3^. The waxy bitumen contained very low abundances of Re and Os (0.02 ppb and 1.34 ppt, respectively), with a ^187^Re/^188^Os ratio of 84.4 and ^187^Os/^188^Os ratio of 0.486. As only a single waxy bitumen specimen was analysed for comparison, no isochron could be constructed to calculate a generation age for its parent crude oil. Nevertheless, the fact that the asphaltites and waxy bitumen, two distinctly different oil types, have clearly different Re-Os abundances and ratios (Table S2) is significant because it suggests they have retained the signatures of their parent oils, despite their exposure to the same marine environment.

### Comparison with the OAE2 osmium isotope record

**Figure 5:**
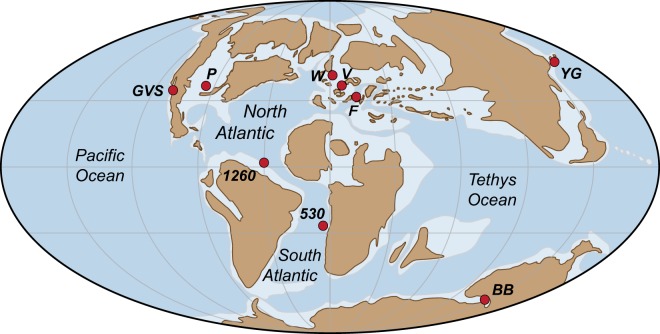
Palaeogeographic map identifying locations of Os isotope records across OAE2 and the location of the Bight Basin. GVS = Great Valley Sequence, P = Portland #1 (GSSP), W = Wunstorf, V = Vocontian Basin, F = Furlo, 1260 = ODP Site 1260, 530 = DSDP Site 530, YG = Yezo Group, BB = Bight Basin. Modified after reference^28^.

**Figure 6:**
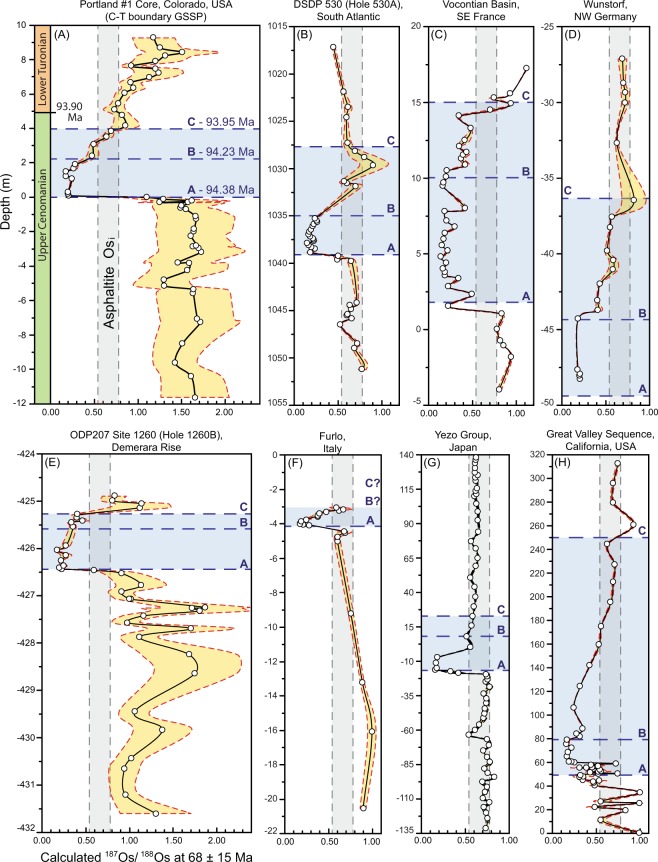
Variation of the calculated ^187^Os/^188^Os compositions at 68 ± 15 Ma with depth for OAE2 sections during the asphaltite generation window. The asphaltite Os_i_ value (0.66 ± 0.12) is shown in grey. Data points and black trendline correspond to the calculated Os_g_ at 68 Ma, while variation in this value due to the uncertainty associated with the generation age is shown in yellow. Areas where asphaltite Os_i_ value = OAE2 section Os_g_ value support potential oil-source correlation. Re-Os data from OAE2 sections after references^26–28^. Location of datums A, B and C labelled in blue within each section are defined by references^27,28,37–41^. The ages of these datums in the Portland #1 core were determined by references^27,42^ and are supported by comparable ages in the Yezo Group^28^. All compiled Re-Os data from these records is listed in Table S6.

Following the generation and expulsion of hydrocarbons, the different abundances of ^187^Re in the source rock and its expelled oil will cause their ^187^Os/^188^Os compositions to gradually diverge as ^187^Re continues to decay to ^187^Os. Therefore, in order to use osmium isotopes to assess the correlation of an oil and a potential source rock, the ^187^Os/^188^Os values at the time of oil generation must be known (i.e. it is the Os_i_ and Os_g_ values that must be compared), as this is the point when the osmium composition of the source rock was imparted directly to the oil. Unfortunately, there is no established osmium isotope record across the Cenomanian-Turonian boundary in the Bight Basin from which to determine an Os_g_ value for direct correlation. However, OAE2 is associated with an ^187^Os/^188^Os anomaly expressed in widely separated palaeogeographic settings^26–28^ (Fig. 5). Therefore, to assess if the asphaltite Os_i_ value (0.66 ± 0.12) is consistent with that of an OAE2 source rock, we calculated the ^187^Os/^188^Os composition for each of these published records across the asphaltite generation window of 68 ± 15 Ma (Fig. 6; Table S6). These Os_g_ profiles preserve an abrupt shift towards non-radiogenic values (~0.2) at the onset of OAE2. The anomalously non-radiogenic conditions are maintained across the A-B stage (94.38–94.23 Ma), prior to a progressive return to radiogenic values within the B-C stage (94.23–93.95 Ma). Despite local variability, the asphaltite Os_i_ value most commonly intersects these OAE2 records during this B-C stage.

## Discussion

To produce a reliable age using Re-Os data three conditions must be met: (1) there must have been no addition or removal of Re or Os, i.e. the samples must remain within a closed system; (2) sufficient variation must exist in the parent/daughter ratios (^187^Re/^188^Os) to produce an isochron; and (3) the samples analysed must contain the same or near-identical initial ^187^Os/^188^Os composition^29^.

A major concern when assessing material released from hydrocarbon seeps is the continued integrity of its Re-Os system despite exposure to formation waters and the marine environment. Studies of weakly to heavily biodegraded crude oils have repeatedly shown that their Re-Os system is not significantly affected by microbial alteration^10,11,13,14^. However, laboratory experiments have demonstrated rapid transfer of Re and Os to liquid oils from an enriched aqueous solution^30^. The authors of this study note that the concentrations of Re and Os used in their experiments were not intended to accurately mimic the natural environment, and that experiments attempting to do so are unlikely to yield meaningful results due to current technical limitations associated with extremely low Re and Os abundances^31^. However, their results demonstrate that Re and Os from formation waters could potentially be imparted to oil in a natural setting. This presents an alternative mechanism for resetting the Re-Os geochronometer and therefore, what the resulting age constrains. If interaction with formation waters occurred in a geologically short timeframe after generation then Re-Os geochronology may still approximate a generation age, although it would actually represent the timing of formation water interaction. Additionally, if the formation waters comprised contemporaneous seawater from when the source rocks were deposited, the resulting ^187^Os/^188^Os composition of the oil would still correlate to the average source rock value. However, if interaction with formation waters occurred much later, the resetting of the geochronometer would constrain the timing of this later alteration. Additionally, if the formation waters comprised migrated groundwater containing Os inherited from sources other than seawater from the time of source rock deposition, then oil-source correlation using the oil’s Os_i_ value would be compromised. Understanding the role played by formation waters in the Re-Os systematics of crude oils in a natural setting is clearly a crucial topic which warrants further investigation. However, it is not practical to assess this competing hypothesis here, as the parent petroleum system of the asphaltites remains undiscovered and is thought to reside within a relatively unexplored basin. Therefore, reliably constraining the impact of groundwater-oil interactions in the subsurface for this system is not currently possible.

Similarly, if the asphaltites sequestered Re and Os from seawater during their time exposed to the marine environment, the required closed system may be compromised, progressively altering the ^187^Os/^188^Os composition of the bitumen towards the present marine value of ~1.06^32^. Such alteration would be expected to increase with continued exposure to seawater. In the case of the asphaltites, their degree of alteration due to both water washing and biodegradation permits an assessment of which specimens have spent the most time in contact with seawater (Fig. 4; Tables S4 and S5). Increasingly degraded asphaltites and interior/exterior comparisons show no systematic trends in their ^187^Re/^188^Os and ^187^Os/^188^Os ratios which could reasonably be attributed to weathering. Furthermore, comparison with a second variety of coastal bitumen from a different petroleum system (waxy bitumen sample W13/007697) demonstrates that the oils retain unique values. Therefore, any alteration of the asphaltites due to their contact with seawater, which contains only trace concentrations of Re and Os, does not appear to have caused a quantifiable disruption of their Re-Os system.

Although our asphaltite sample suite displays sufficient variation in ^187^Re/^188^Os to produce a meaningful isochron (Fig. 3), the mechanism behind this variation is not fully understood. As discussed above, variation may be driven by interaction with formation waters^30^, or because the asphaltites analysed in this study were collected over the course of three years, and likely represent products of continuous, low intensity seepage. Therefore, the variation could also be attributable to the prolonged duration (>1 Myr) of bitumen generation and expulsion when the chronometer is initially reset^12^.

The MSWD of the revised regression (Fig. 3B) is low (<1), owing to the tight clustering of the analysed samples. Although an improvement from the regression of all asphaltite Re-Os data, the revised regression also yields a date that is still relatively imprecise (68 ± 15 Ma, 22%). Such a spread in a Re-Os generation age is not abnormal^11,13,14^, and likely the result of prolonged generation over millions of years, combined with variation in the initial ^187^Os/^188^Os composition of the source unit^11,12,14^. Potential mixing of variable ^187^Os/^188^Os values across a source rock may be particularly applicable to oils and bitumen derived from OAE2 deposits, given the extent of variability at metre to centimetre scale across the interval (Fig. 6). Progressive generation across the OAE2 interval, potentially augmented by hydrocarbons expelled from thermally mature, pre- or post-OAE2 organic matter, would likely create a spread in the Os_i_ values of the resulting oil, thereby reducing the precision of the final Re-Os age.

Assessment of Cretaceous potential source rocks in the Bight Basin using a two-dimensional basin model suggests that a Cenomanian-Turonian source interval would have generated and expelled hydrocarbons from the mid-Campanian (~74 Ma) to the present^20^. A previous one-dimensional model also supports generation starting from approximately the mid-Campanian^33^. The age determined by the Re-Os systematics of the asphaltites is consistent with these models, supporting the interpretation that this age represents the timing of generation and that the parent petroleum system incorporates an OAE2 source rock.

Previous studies conclude that the Os_i_ value of an oil is inherited directly from its source rock and may therefore aid oil-source correlations^9–11,13–15^. The comparison of the asphaltite Os_i_ with the Os_g_ profiles of other, well-studied examples of OAE2 may provide preliminary interpretations regarding the nature of the asphaltite source, as the event preserves globally reproducible trends (Fig. 6). In no case do the asphaltites appear likely to be derived exclusively from organic matter deposited during the onset of OAE2, as the Os_i_ (0.66 ± 0.12) is notably more radiogenic than the calculated Os_g_ of the A-B interval (~0.2; Fig. 6). However, in most examples, the asphaltite Os_i_ range is consistent with the Os_g_ values in the latter section of OAE2 (interval B-C), as well as those of the pre- and post-OAE2 intervals in certain regions (Fig. 6).

Although the asphaltites are thought to originate from an OAE2 source rock^17,20^, no exploratory or stratigraphic drilling within the Bight Basin has reached the Cenomanian-Turonian boundary to provide confirmation. A recent attempt to assess the potential for preservation of an OAE2 interval within the Bight Basin by the International Ocean Discovery Program (IODP) Expedition 369 was unable to reach to the necessary depth, due to a hole deviation of ~26° from vertical during coring operations^34^. The fact that organic-rich lithofacies associated with OAE2 are yet to identified and sampled does not preclude the possibility that other source rocks which may have been deposited in the offshore Bight Basin due to local anoxic conditions arising from its palaeogeographic location within a narrow re-entrant of the Tethys Ocean (Fig. 5). This scenario would similarly satisfy the requirements defined by their geochemistry^2,17^. Multiple alternative marine intervals with high source potential are also inferred from the late Aptian to Santonian in the Bight Basin based on the tectonostratigraphic framework defined by data from wells drilled in the proximal regions of the basin^21,33^. However, the existence of these putative basinward source intervals is similarly yet to be confirmed.

While the OAE2 interval is characterised by an abrupt non-radiogenic shift in ^187^Os/^188^Os values (Fig. 6), the global record also demonstrates regional variability driven by differential inputs of osmium from local continental weathering and submarine volcanism^27,28^. The extent to which such inputs may have impacted the ^187^Os/^188^Os ratio across the Cenomanian-Turonian boundary interval in the Bight Basin remains unknown until a local record becomes available. Meanwhile, the lack of relevant analytical data on potential source rocks in the distal reaches of the basin precludes definitive asphaltite-source rock correlation. Such gaps in the knowledge of frontier basins are common. The uncertainties inherent in our initial interpretation of the Os isotope signatures of these Australian asphaltites will be reduced as understanding of the geology and geochemistry of the Bight Basin improves with further exploration.

## Conclusions

Through the application of Re-Os radiometric dating to heavy asphaltic crude oil released from natural seafloor seepage we have demonstrated that the technique may be applied to constrain the timing of hydrocarbon generation and potential source intervals within offshore sedimentary basins. The Re-Os systematics of crude oils stranding in coastal environments may therefore have widespread application to the identification of their respective offshore hydrocarbon systems. The ability to inform or validate the timing of generation determined from basin models provides a new approach to reducing the uncertainty and risk associated with petroleum exploration in frontier basins, particularly in deepwater settings where the cost of exploratory drilling is extremely high. Our findings also highlight the potential use of Os isotopes in correlating oils to their parent source rocks at high resolution, particularly those deposited during OAE2 which exhibit highly anomalous ^187^Os/^188^Os values. However, such correlations may be affected by inherent local variability in the marine osmium isotope record and hence wherever possible should use data from the proposed source rock in the basin of origin, rather than the global record.

## Materials and Methods

### Re-Os isotope data

All Re-Os sample preparation and isotopic analyses were conducted at the Laboratory for Source Rock Geochronology and Geochemistry and Arthur Holmes Laboratory at Durham University (UK). Samples were prepared for Re-Os analysis using published methods^16^. Approximately 200 mg of whole oil sample from each asphaltite was dissolved in a minimum volume of chloroform (CHCl_3_) and loaded into a Carius tube, with the solvent subsequently evaporated at 65 °C. All samples were then spiked with a mixed tracer solution containing a known amount of ^185^Re and ^190^Os. The tracer solution was homogenised with the sample in the Carius tube using inverse *aqua regia* (3 mL of ~12 N HCl, 6 mL of 16 N HNO_3_) and heated at 220 °C for 48 hours. Osmium was subsequently extracted and purified using CHCl_3_ solvent extraction and microdistillation. The Re-bearing *aqua regia* was dried on a hot plate at 80 °C overnight and then purified by NaOH-acetone solvent extraction and anion chromatography.

The purified Re and Os were loaded onto Ni and Pt wire filaments, respectively, and analysed by Negative Thermal Ionization Mass Spectrometry (NTIMS) on a Thermo Scientific TRITON mass spectrometer. The total procedural blanks were 2.4 ± 1.0 pg/g for Re and 0.15 ± 0.05 pg/g for Os, with a ^187^Os/^188^Os of 0.25 ± 0.05 (n = 4). The uncertainty for all Re-Os data was determined using full error propagation of uncertainties. The Re-Os data were regressed using *Isoplot V4*.*15*^24^ and a ^187^Re decay constant of 1.666 × 10^−11^ a^−1^ ^35^.

### Re-Os data deviation assessment

Data points responsible for introducing significant uncertainty in the regression of all the available data (Fig. 3A) were identified by determining the percentage of deviation between the observed ^187^Os/^188^Os composition in each sample compared to the predicted value defined by the regression described by Equation (1). The calculated value for each sample was obtained using the isochron equation listed as Equation (2), where λ is the decay constant for ^187^Re and *t* is the age in years, using values defined by the regression of all of data points (i.e. where *t* = 68 Myr and ^187^Os/^188^Os_initial_ = 0.63). Samples with a deviation of >2% from the initial regression were excluded for the revised regression (Fig. 3B).

1 $${\rm{Deviation}}( \% )=({}^{187}{\rm{O}}{\rm{s}}{/}^{188}{{\rm{Os}}}_{{\rm{measured}}}\,-\,{}^{187}{\rm{O}}{\rm{s}}{/}^{188}{{\rm{Os}}}_{{\rm{calculated}}{\rm{from}}{\rm{data}}{\rm{best}}-{\rm{fit}}{\rm{initial}}})\times 100$$

2 $${}^{187}{\rm{O}}{\rm{s}}/{}^{188}{\rm{O}}{\rm{s}}={}^{187}{\rm{O}}{\rm{s}}/{}^{188}{\rm{O}}{{\rm{s}}}_{{\rm{initial}}}+{}^{187}{\rm{R}}{\rm{e}}/{}^{188}{\rm{O}}{\rm{s}}\times ({{\rm{e}}}^{{\rm{\lambda }}t}\,-\,1)$$

### Calculation of OAE2 osmium composition during the asphaltite generation window

The ^187^Os/^188^Os composition of each OAE2 section during the asphaltite generation window of 68 ± 15 Ma was calculated using Equation (2) and the composition of each individual data point is listed in Table S6.

### Whole-oil gas chromatography-mass spectrometry

All sample preparation and analyses were conducted in the Biogeochemistry Laboratory at the University of Adelaide. A sub-sample (≤200 mg) was removed from each bitumen specimen and dissolved in 10 mL of dichloromethane/methanol (93:7, v:v). An aliquot of this solution was used for whole-oil GC-MS. Analyses of the samples collected in 2014 and 2015 were performed on an Agilent 6890 gas chromatograph interfaced with a 5973 N MSD (electron energy 70 eV) and tuned using automatic setup parameters for each sequence. Chromatography was carried out on an Agilent HP-5MS fused silica column (30 m × 0.25 mm i.d. × 0.25 µm film thickness), using either a split (50 mL/min) or splitless injection mode, the latter employed if split injection data showed low responses. The oven was held at an initial temperature of 50 °C for 1 min, followed by heating at 8 °C/min to 300 °C. The carrier gas was helium at a flow rate of 1 mL/minute. GC-MS analyses of the samples collected in 2016 were performed on an Agilent 7890B gas chromatograph interfaced with a 5977B MSD (electron energy 70 eV) and tuned using automatic setup parameters for each sequence. Chromatography was undertaken on a J&W DB-5MS fused silica column (30 m × 0.25 mm i.d. × 0.25 µm film thickness), using the same operating conditions as employed for the 2014 and 2015 samples. In both cases, whole-oil GC-MS was conducted in the scan mode (scan interval 45–500 AMU at approx. 3 scans/sec). All data were processed using *MSD ChemStation* (Agilent Technologies, Inc.).

### Isolation of saturated, aromatic and asphaltene fractions

The saturated and aromatic hydrocarbon fractions were separated from 20 mg aliquots of whole bitumen using silica gel liquid chromatography^36^. The recovered saturated hydrocarbons were stored in 2 mL chromatography vials and the aromatics in 2 mL amber glass vials to ensure minimal degradation by ultraviolet light. The asphaltene fraction of each sample was separated by adding an excess of *n*-pentane to bulk bitumen sub-samples (0.3–0.7 g) dissolved in a mixture of dichloromethane and methanol (93:7). The dissolved maltene fraction was then removed using a Pasteur pipette, allowing the precipitated asphaltene fraction to be dried and weighed.

### Selected ion monitoring gas chromatography-mass spectrometry (SIM GC-MS)

GC-MS analysis of the saturated hydrocarbon fractions for sterane and hopane degradation assessment was performed on an Agilent 7890 A gas chromatograph interfaced with a 5975 C MSD (electron energy 70 eV) tuned using automatic setup parameters on the day of the analysis. Chromatography was carried out on a J&W DB-5MS fused silica column (60 m × 0.25 mm i.d. × 0.25 µm film thickness). A 1 μL aliquot of the saturates fraction was injected into the split/splitless injector at 300 °C operating in splitless mode. After being held at an initial temperature of 40 °C for 2 minutes, the oven was heated at 20 °C/minute to 200 °C and then ramped to 310 °C at 2 °C/minute. The carrier gas was helium at a flow rate of 1.5 mL/min.

GC-MS analyses of the aromatic fractions to determine alteration to water-soluble compounds was performed on a Perkin Elmer SQ8T/680 Clarus gas chromatograph. Chromatography was carried out on a Perkin Elmer PE-5MS fused silica column (30 m × 0.25 mm i.d. × 0.25 µm film thickness). A 1 μL aliquot of the aromatics fraction was injected into the split/splitless injector at 300 °C operating in splitless mode. The oven temperature was held at 50 °C for 1 min., followed by heating at 8 °C/min to 300 °C. Helium was the carrier gas at a flow rate of 1 ml/min. Data were acquired in the scan mode (Scan 45:500AMU at approx. 3 scans/sec).

## Supplementary information

Supplementary Information

## Data Availability

All data needed to assess the conclusions of this paper are included in the paper and/or Supplementary Materials. Any additional information regarding samples is available on request.
