# Supplementary material for: Remotely constraining the temporal evolution of offshore oil systems
Source: Sci Rep. 2019 Feb 4;9:1327. doi: 10.1038/s41598-018-37884-x (PMC6362295; doi:10.1038/s41598-018-37884-x)
Supplement: Supplementary file 1 — Supplementary Information [file 41598_2018_37884_MOESM1_ESM.pdf]

Supplementary Information

Remotely constraining the temporal evolution of offshore oil systems

Alexander J. Corrick[1*], David Selby[2,3], David M. McKirdy[1], Philip A. Hall[1], Se Gong[4], Christine Trefry[5] and Andrew S. Ross[5]

* Corresponding author: alexander.corrick@adelaide.edu.au

[1] Department of Earth Sciences, School of Physical Sciences, University of Adelaide, SA 5005, Australia

[2] Department of Earth Sciences, Durham University, Durham DH1 3LE, UK

[3] State Key Laboratory of Geological Processes and Mineral Resources, School of Earth Resources, China University of Geosciences, Wuhan, 430074 Hubei, China

[4] Energy, CSIRO, North Ryde, NSW 2113, Australia

[5] Energy, CSIRO, Kensington, WA 6151, Australia

Summary:

Table S1. Sample collection details.

Table S2. Re-Os data.

Table S3. Asphaltites Re-Os regression deviation assessment.

Table S4. Selected geochemical parameters and assigned degradation levels for analysed samples

Table S5. Degradation level classification criteria

Table S6. Re-Os data compiled for OAE2 sections and calculated Os_g values across asphaltite generation window (68 ± 15 Ma).

Table S1. Sample collection details. Co-ordinates use WGS84 datum and unless otherwise stated have a maximum error of ± 5m.

Sample	Stranding Location	Latitude	Longitude	Date Collected	Weight (g)
Asphaltites					
W13/007476	Cactus Beach	-32.0739	132.9761	10-Nov-14	813
W13/007477	Cactus Beach	-32.0689	132.9706	10-Nov-14	219
W13/007507	Waitpinga Beach	-35.6362	138.5061	22-Nov-14	601
W13/007516	The Granites	-36.6517	139.8520	23-Nov-14	170
W13/007668	Tractor Beach	-32.8675	134.1178	21-Sep-15	25
W13/007671	Cactus Beach	-32.5328	133.8636	23-Sep-15	276
W13/007672	Avoid Bay*	-34.66	135.49	31-May-15	1357
W13/007742	Greenly Beach*	-34.46	135.36	22-Aug-15	392
W13/007764	Eyre Well	-31.4651	131.1338	26-Sep-15	429
W13/007845	Sheringa Beach	-33.8744	135.1781	11-Oct-16	500
W13/007976	Waitpinga Beach	-35.6374	138.5103	19-Oct-16	3188
Waxy bitumen (for comparison)					
W13/007697	Avoid Bay*	-34.64	135.35	13-Jul-15	35

* = Sample donated from private collection. Location co-ordinates are approximate.

Table S2: Re-Os data.

Sample	Re (ppb)	±	Os (ppt)	±	^{192}Os (ppt)	±	^{187}Re / ^{188}Os	±	^{187}Os / ^{188}Os	±	Rho	% Re Blank	% ^{187}Os Blank	% ^{188}Os Blank	Osi (@ 74 Ma)	Osi (@ 53 Ma)	Osi (@ 68 Ma)	Osi (@ 83 Ma)	±
Asphaltites																			
W13/007476 (exterior)	3.11	0.01	33.5	0.5	12.1	0.3	514.1	14.7	1.263	0.046	0.776	1.2	1.7	8.7	0.63	0.81	0.68	0.55	0.07
W13/007477 (exterior)	3.09	0.01	32.5	0.5	11.7	0.3	527.7	15.2	1.280	0.047	0.776	1.2	1.7	8.8	0.63	0.81	0.68	0.55	0.07
W13/007477 (interior)	2.95	0.01	32.6	0.5	11.7	0.3	502.5	14.2	1.288	0.046	0.773	1.2	1.6	8.4	0.67	0.84	0.72	0.59	0.07
W13/007507 (interior)	2.65	0.01	28.0	0.4	10.1	0.3	521.4	15.4	1.244	0.045	0.798	0.5	0.8	4.0	0.60	0.78	0.65	0.52	0.07
W13/007507 (interior replicate)	2.15	0.01	24.6	0.6	8.8	0.5	483.7	24.8	1.265	0.083	0.771	0.7	0.4	2.1	0.67	0.84	0.72	0.60	0.09
W13/007516 (exterior)	3.01	0.01	31.7	0.5	11.4	0.3	526.3	15.3	1.303	0.047	0.781	1.2	1.7	8.9	0.65	0.84	0.71	0.58	0.07
W13/007516 (interior)	3.00	0.01	30.3	0.5	10.9	0.3	547.8	16.0	1.285	0.047	0.776	1.1	1.7	8.9	0.61	0.80	0.66	0.53	0.07
W13/007668 (interior)	3.08	0.01	32.3	0.6	11.6	0.3	528.6	15.5	1.278	0.068	0.539	1.2	1.8	9.2	0.63	0.81	0.68	0.55	0.09
W13/007671 (exterior)	2.99	0.01	32.4	0.5	11.6	0.3	512.6	15.1	1.291	0.048	0.776	1.2	1.8	9.1	0.66	0.84	0.71	0.58	0.07
W13/007671 (interior)	4.02	0.01	43.1	0.6	15.6	0.4	513.6	12.9	1.233	0.040	0.756	0.3	0.5	2.7	0.60	0.78	0.65	0.52	0.06
W13/007672 (interior)	2.68	0.01	36.4	0.5	13.3	0.3	400.5	10.3	1.122	0.037	0.758	0.4	0.6	2.8	0.63	0.77	0.67	0.57	0.05
W13/007742 (interior)	2.85	0.01	30.3	0.5	10.9	0.3	521.5	16.2	1.299	0.050	0.794	1.4	2.0	10.4	0.66	0.84	0.71	0.58	0.08
W13/007764 (exterior)	3.07	0.02	32.2	0.5	11.5	0.3	529.6	15.3	1.294	0.047	0.772	1.1	1.7	8.7	0.64	0.83	0.69	0.56	0.07
W13/007764 (interior)	3.04	0.01	33.0	0.5	11.9	0.3	508.0	13.8	1.219	0.042	0.775	0.4	0.7	3.3	0.59	0.77	0.64	0.52	0.06
W13/007845 (interior)	2.89	0.01	31.2	0.5	11.2	0.3	515.5	15.1	1.296	0.048	0.783	1.2	1.8	9.2	0.66	0.84	0.71	0.58	0.07
W13/007976 (interior)	2.59	0.01	30.1	0.4	10.9	0.3	470.7	13.0	1.176	0.041	0.780	0.4	0.7	3.5	0.60	0.76	0.64	0.52	0.06
Waxy Bitumen (for comparison)																			
W13/007697 (interior)	0.02	0.01	1.34	0.3	0.5	0.3	84.4	60.8	0.486	0.279	0.544	65.0	16.5	32.3	N/A	N/A	N/A	N/A	N/A

Individual ratio uncertainties are given at the 2σ level.

Rho = error correlation value between $^{187}Re/^{188}Os$ and $^{187}Os/^{188}Os$

Os_i uncertainties are determined through full propagation of uncertainties in Re and Os mass spectrometry, spike, blanks and std Rd and Os isotopic values.

Table S3. Asphaltite Re-Os deviation assessment.

Sample	^{187}Re / ^{188}Os	±	^{187}Os / ^{188}Os	±	^{187}Os / ^{188}Os calculated from data best-fit initial	Difference	% deviation from regression #1	Comments
W13/007476 (exterior)	514.1	14.7	1.263	0.046	1.247	0.017	1.3	
W13/007477 (exterior)	527.7	15.2	1.280	0.047	1.263	0.017	1.3	
W13/007477 (interior)	502.5	14.2	1.288	0.046	1.233	0.055	4.4	>2% deviation. Excluded from regression #2
W13/007507 (interior)	521.4	15.4	1.244	0.045	1.256	-0.012	-0.9	
W13/007507 (interior replicate)	483.7	24.8	1.265	0.083	1.210	0.054	4.5	>2% deviation. Excluded from regression #2
W13/007516 (exterior)	526.3	15.3	1.303	0.047	1.262	0.042	3.3	>2% deviation. Excluded from regression #2
W13/007516 (interior)	547.8	16.0	1.285	0.047	1.287	-0.003	-0.2	
W13/007668 (interior)	528.6	15.5	1.278	0.068	1.264	0.013	1.0	
W13/007671 (exterior)	512.6	15.1	1.291	0.048	1.245	0.046	3.7	>2% deviation. Excluded from regression #2
W13/007671 (interior)	513.6	12.9	1.233	0.040	1.246	-0.013	-1.1	
W13/007672 (interior)	400.5	10.3	1.122	0.037	1.111	0.011	1.0	
W13/007742 (interior)	521.5	16.2	1.299	0.050	1.256	0.043	3.4	>2% deviation. Excluded from regression #2
W13/007764 (exterior)	529.6	15.3	1.294	0.047	1.266	0.028	2.3	>2% deviation. Excluded from regression #2
W13/007764 (interior)	508.0	13.8	1.219	0.042	1.240	-0.020	-1.6	
W13/007845 (interior)	515.5	15.1	1.296	0.048	1.249	0.047	3.8	>2% deviation. Excluded from regression #2
W13/007976 (interior)	470.7	13.0	1.176	0.041	1.195	-0.019	-1.6	

Isochron equation for regression #1 (all data): $^{187}Os/^{188}Os_{\text{calculated from data best-fit initial}} = 0.63 + 0.0012 \times {}^{187}Re/^{188}Os$

Samples which deviate > 2% from regression #1 were excluded from regression #2.

Table S4. Selected geochemical parameters and assigned degradation levels for analysed samples.

Sample	Asphaltene content (%)	Relative degradation level	Soft vs. Brittle*	Lightest *n*-alkane preserved	Pr / Ph	MNR (*m/z* 142)	DNR-1 (*m/z* 156)	Ts / (Ts+Tm)	C_{29} / C_{30} αβ hopane	27 : 28 : 29 ααα 20*R* steranes (*m/z* 217)	27: 28 : 29 αββ (20*R*+*S*) steranes (*m/z* 218)	C_{27} Dia / (Dia + Regular)	C_{35} homohopane index (%)
Asphaltites													
W13/007476	53	Moderate	Brittle	10	1.3	0.81	2.39	0.41	0.60	43 : 20 : 38	38 : 25 : 37	0.42	7.6
W13/007477	56	Moderate	Brittle	10	1.1	0.72	2.03	0.40	0.59	47 : 18 : 36	38 : 24 : 37	0.44	7.4
W13/007507	54	Moderate	Brittle	10	1.2	0.98	2.43	0.40	0.65	40 : 27 : 33	39 : 25 : 36	0.43	7.4
W13/007516	55	Moderate	Brittle	10	1.3	0.72	2.46	0.38	0.66	44 : 19 : 37	38 : 25 : 37	0.42	8.0
W13/007668	59	High	Brittle	14	1.1	0.30	1.20	0.38	0.67	37 : 27 : 36	37: 26 : 37	0.45	7.7
W13/007671	54	Moderate	Brittle	9	1.1	0.72	2.31	0.41	0.65	37 : 23 : 39	37 : 25 : 38	0.42	7.9
W13/007672	54	Moderate	Brittle	9	1.2	0.77	2.31	0.38	0.65	43 : 20 : 37	39 : 25 : 36	0.43	8.4
W13/007742	54	Moderate	Brittle	10	1.0	0.78	1.56	0.39	0.64	39 : 23 : 38	39 : 23 : 38	0.45	8.3
W13/007764	56	Moderate	Brittle	9	1.2	0.69	2.41	0.35	0.61	38 : 25 : 37	36 : 24 : 40	0.42	10.9
W13/007845	51	Low	Soft	< 8‡	1.2	0.83	2.58	0.37	0.64	39 : 27 : 34	39 : 26 : 35	0.46	7.2
W13/007976	51	Low	Soft	< 8‡	1.2	0.93	2.98	0.41	0.62	39 : 26 : 36	38 : 25 : 37	0.45	7.6
Waxy bitumen													
W13/007697	32	Moderate / High	Soft†	12	1.8	0.84§	5.84§	0.60	0.67	39 : 21 : 40	42 : 22 : 37	0.47	4.7

* At the time of initial sub-sampling. Asphaltites devolatilise their low-molecular-weight hydrocarbons during storage and will eventually become brittle like their weathered counterparts.

† Waxy bitumen remain semi-solid and will only develop a brittle exterior if extremely degraded.

‡ *n*-alkanes lighter than C_8 (octane) were outside the detection limit of the whole-oil GC-MS analysis.

§ Compounds present in low abundance.

MN = Methylnaphthalene ratio = 2-MN / 1-MN

DNR = Dimethylnaphthalene ratio = (2,6-DMN + 2,7-DMN) / 1,5-DMN

C_{27} Dia / Dia + Regular = C_{27} βα (20*S*+*R*) diacholestanes / (C_{27} βα (20*S*+*R*) diacholestanes + C_{27} ααα (20*S*+*R*) cholestanes

C_{35} homohopane index (%) = (C_{35} αβ (*S*+*R*) homohopanes / $C_{31\text{-}35}$ αβ (*S*+*R*) homohopanes) x 100

Table S5. Degradation level classification criteria. Classification for each sample is based on the characteristics of the interior sub-sample.

Degradation Level	Physical characteristics (soft / brittle)*	*n*-alkanes <C_{10}	*n-alkanes* <C_{14}	Water-soluble compounds (e.g. alkylnaphthalenes)	Steranes and hopanes
Low	Soft	Preserved/Altered	Preserved	Preserved	Preserved
Moderate	Brittle	Altered/Lost	Preserved/Altered	Preserved / Mild Alteration	Preserved
High	Brittle	Lost	Lost	Significant alteration	Potential minor alteration to the C_{30} αβ hopane and C_{27} steranes

* Only asphaltites become brittle due to weathering. The waxy bitumen used for comparison remains semi-solid despite moderate degradation.

Table S6. Compiled Re-Os data for OAE2 sections and calculated Os_g values for asphaltite generation window (68 ± 15 Ma).

Sample ID	Reference	Depth (m)	Re (ppb)	±	Os (ppt)	±	^{187}Re / ^{188}Os	±	^{187}Os / ^{188}Os	±	^{192}Os (ppt)	Os_g (@ 53 Ma)	Os_g (@ 68 Ma)	Os_g (@83 Ma)	±
Portland #1 Core, Colorado, USA (Fig. 6A)															
AD117-09	(27)	9.30	47.5	0.15	298.4	1.19	983.5	4.2	2.295	0.008	96.0	1.427	1.181	0.935	0.004
AD115-09	(27)	8.71	49.4	0.16	290.3	1.17	1071.7	4.7	2.472	0.008	91.8	1.525	1.257	0.989	0.004
AD114-09	(27)	8.46	75.2	0.24	303.3	1.32	1714.1	7.5	3.453	0.011	87.3	1.939	1.510	1.081	0.004
AD112-09	(27)	8.25	55.9	0.18	277.8	1.19	1306.5	5.9	2.794	0.010	85.1	1.640	1.313	0.986	0.004
AD111-09	(27)	7.92	69.5	0.22	399.2	1.46	1093.0	4.3	2.442	0.007	126.6	1.477	1.203	0.930	0.004
AD110-09	(27)	7.65	1.3	0.01	14.6	0.35	500.4	26.2	1.494	0.084	5.1	1.052	0.927	0.801	0.055
AD109-09	(27)	7.49	52.5	0.17	296.8	1.28	1111.8	5.0	2.466	0.009	93.9	1.484	1.206	0.928	0.004
AD108-09	(27)	7.25	66.3	0.21	366.2	1.42	1146.5	4.7	2.540	0.008	115.0	1.527	1.241	0.954	0.004
AD107-09	(27)	6.98	50.9	0.16	323.7	1.25	964.7	4.1	2.229	0.007	104.9	1.376	1.135	0.894	0.004
AD106-09	(27)	6.69	8.3	0.03	90.9	0.65	516.6	5.3	1.504	0.017	31.8	1.048	0.919	0.789	0.011
AD105-09	(27)	6.37	12.6	0.04	129.2	0.77	558.6	4.3	1.583	0.013	44.8	1.090	0.950	0.810	0.008
AD104-09	(27)	6.07	2.0	0.01	27.4	0.45	414.2	13.5	1.317	0.050	9.8	0.951	0.848	0.744	0.034
AD103-09	(27)	5.47	5.3	0.02	88.5	0.77	327.4	4.5	1.143	0.020	32.2	0.854	0.772	0.690	0.014
AD102-09	(27)	5.11	17.7	0.06	189.0	0.91	521.2	3.1	1.325	0.009	67.5	0.865	0.734	0.604	0.005
AD101-09	(27)	4.80	7.2	0.03	98.0	0.74	407.4	4.6	1.282	0.017	35.2	0.923	0.821	0.719	0.011
AD99-09	(27)	4.17	35.0	0.11	319.0	1.08	627.9	2.6	1.572	0.005	110.8	1.018	0.861	0.704	0.003
AD98-09	(27)	3.85	4.8	0.02	111.6	0.75	229.3	2.5	0.955	0.012	41.6	0.752	0.695	0.637	0.010
AD96-09	(27)	3.52	3.9	0.02	77.5	0.63	267.7	3.7	0.948	0.016	28.9	0.712	0.645	0.578	0.012
AD95-09	(27)	3.10	4.6	0.02	134.2	0.92	175.9	2.1	0.697	0.011	51.6	0.542	0.498	0.454	0.008
AD93-09	(27)	2.40	5.4	0.02	100.9	0.67	278.8	3.1	0.792	0.011	38.3	0.546	0.476	0.406	0.006
AD90-09	(27)	1.90	17.0	0.06	532.6	1.41	160.2	0.7	0.468	0.002	210.5	0.327	0.286	0.246	0.001
AD88-09	(27)	1.67	16.9	0.06	570.4	1.47	148.6	0.7	0.438	0.002	226.3	0.307	0.270	0.232	0.001
AD87-09	(27)	1.50	4.7	0.02	670.3	3.46	34.1	0.4	0.210	0.003	273.7	0.180	0.171	0.162	0.003
AD84-09	(27)	1.25	4.1	0.02	621.4	4.12	32.0	0.5	0.210	0.004	253.8	0.181	0.173	0.165	0.004
AD83-09	(27)	1.10	3.7	0.02	213.8	1.52	86.5	1.2	0.344	0.007	85.8	0.268	0.246	0.224	0.005
AD68-09	(27)	0.30	1.9	0.01	592.8	5.62	15.3	0.3	0.216	0.006	241.9	0.202	0.199	0.195	0.007
AD69-09	(27)	0.20	18.6	0.06	1116.0	2.51	81.9	0.4	0.296	0.001	450.8	0.224	0.203	0.183	0.001
AD70-09	(27)	0.10	35.4	0.12	1716.0	2.90	102.1	0.4	0.328	0.001	690.3	0.238	0.213	0.187	0.001
AD71-09	(27)	0.00	111.9	0.55	554.6	1.68	1280.3	6.6	2.558	0.005	173.9	1.427	1.106	0.786	0.003
AD72-09	(27)	-0.10	58.3	0.19	324.1	1.38	1146.2	5.0	2.591	0.009	101.3	1.578	1.292	1.005	0.005
AD73-09	(27)	-0.20	66.7	0.22	262.4	1.26	1788.0	8.5	3.646	0.013	74.3	2.067	1.619	1.172	0.005

Sample ID	Reference	Depth (m)	Re (ppb)	±	Os (ppt)	±	^{187}Re / ^{188}Os	±	^{187}Os / ^{188}Os	±	^{192}Os (ppt)	Os_g (@ 53 Ma)	Os_g (@ 68 Ma)	Os_g (@83 Ma)	±
AD74-09	(27)	-0.30	51.8	0.17	320.5	1.25	1009.9	4.3	2.397	0.008	102.1	1.505	1.253	1.000	0.004
AD75-09	(27)	-0.30	71.8	0.24	301.0	1.35	1646.9	7.4	3.447	0.011	86.7	1.992	1.581	1.168	0.005
AD76-09	(27)	-0.50	59.0	0.19	290.0	1.27	1350.9	6.0	3.034	0.010	86.8	1.840	1.502	1.164	0.005
AD77-09	(27)	-0.60	72.7	0.23	371.9	1.48	1288.4	5.2	2.951	0.008	112.2	1.813	1.491	1.169	0.005
AD78-09	(27)	-0.70	70.7	0.23	323.0	1.42	1481.7	6.4	3.232	0.011	94.9	1.923	1.552	1.182	0.005
AD118-09	(27)	-1.10	87.3	0.28	346.6	1.49	1776.5	7.5	3.678	0.011	97.8	2.109	1.664	1.220	0.002
AD119-09	(27)	-1.25	84.3	0.27	332.2	1.47	1792.6	7.7	3.702	0.011	93.6	2.118	1.670	1.221	0.002
AD79-09	(27)	-1.84	82.4	0.27	326.5	1.43	1775.8	7.6	3.660	0.011	92.3	2.092	1.647	1.203	0.005
AD120-09	(27)	-1.98	67.7	0.22	269.4	1.27	1766.0	8.1	3.644	0.013	76.3	2.084	1.642	1.200	0.003
AD121-09	(27)	-2.26	58.2	0.19	251.0	1.23	1597.1	7.7	3.423	0.013	72.5	2.012	1.613	1.213	0.003
AD80-09	(27)	-2.81	83.5	0.27	328.7	1.71	1796.2	8.3	3.711	0.016	95.7	2.124	1.675	1.226	0.006
AD122-09	(27)	-2.87	70.0	0.23	295.3	1.36	1642.9	7.4	3.492	0.012	84.7	2.041	1.630	1.219	0.003
AD123-09	(27)	-2.95	67.2	0.22	256.8	1.32	1866.9	9.2	3.817	0.015	71.6	2.168	1.701	1.233	0.003
AD124-09	(27)	-3.18	76.3	0.25	276.7	1.80	1999.3	13.0	3.993	0.025	75.9	2.227	1.727	1.227	0.004
AD81-09	(27)	-3.79	91.0	0.29	391.3	1.58	1576.1	6.3	3.246	0.009	114.8	1.854	1.460	1.065	0.004
AD126-09	(27)	-3.82	125.7	0.41	497.0	2.25	1770.4	7.8	3.598	0.012	141.2	2.034	1.591	1.148	0.002
AD128-09	(27)	-4.25	84.4	0.27	333.4	1.87	1768.1	9.9	3.576	0.018	94.9	2.014	1.571	1.129	0.003
AD82-09	(27)	-4.80	69.7	0.22	365.6	1.51	1226.8	5.1	2.692	0.009	113.1	1.608	1.301	0.994	0.004
AD130-09	(27)	-5.16	85.1	0.27	400.9	2.28	1389.3	7.6	2.878	0.016	121.8	1.651	1.303	0.956	0.003
AD132-09	(27)	-5.36	68.1	0.22	305.4	2.52	1529.8	12.3	3.375	0.034	88.6	2.024	1.641	1.258	0.006
AD133-09	(27)	-5.59	73.5	0.24	299.3	1.91	1715.9	10.7	3.582	0.022	85.2	2.067	1.637	1.208	0.004
501.61	(27)	-7.09	90.0	0.29	366.6	2.40	1724.6	11.8	3.638	0.024	103.8	2.114	1.683	1.251	0.004
AD134-09	(27)	-7.26	82.7	0.27	319.1	1.90	1850.8	10.8	3.821	0.020	88.9	2.186	1.723	1.260	0.004
505.9	(27)	-8.53	64.6	0.21	328.2	2.15	1301.8	9.5	2.986	0.021	98.7	1.836	1.510	1.185	0.005
509.5	(27)	-9.65	45.0	0.15	278.4	1.94	1029.1	8.6	2.597	0.022	87.0	1.688	1.431	1.173	0.006
512	(27)	-10.43	56.8	0.19	271.3	2.04	1415.7	12.4	3.215	0.029	79.9	1.964	1.610	1.256	0.006
516	(27)	-11.65	52.2	0.17	227.6	1.85	1583.0	15.9	3.453	0.034	65.6	2.055	1.659	1.263	0.007

DSDP Site 530, Hole 530A, South Atlantic (Fig. 6B)

Sample ID	Reference	Depth (m)	Re (ppb)	±	Os (ppt)	±	^{187}Re / ^{188}Os	±	^{187}Os / ^{188}Os	±	^{192}Os (ppt)	Os_g (@ 53 Ma)	Os_g (@ 68 Ma)	Os_g (@83 Ma)	±
AD80-10	(27)	1017.18	0.2	0.01	14.3	0.38	70.3	6.00	0.530	0.041	5.6	0.468	0.450	0.433	0.024
AD86-10	(27)	1021.88	11.6	0.04	682.2	3.05	87.5	0.62	0.665	0.006	263.2	0.588	0.566	0.544	0.003
AD88-10	(27)	1023.53	10.7	0.04	331.6	1.79	169.9	1.41	0.808	0.008	125.7	0.658	0.616	0.573	0.004
AD90-10	(27)	1024.58	2.8	0.01	215.5	1.72	66.0	0.97	0.671	0.013	83.1	0.613	0.597	0.580	0.007
AD92-10	(27)	1027.29	35.4	0.13	1025.7	5.04	181.0	1.34	0.816	0.007	388.5	0.656	0.611	0.566	0.003
AD94-10	(27)	1028.16	156.2	0.51	2385.9	6.56	355.6	1.38	1.100	0.003	874.1	0.786	0.697	0.608	0.001

Sample ID	Reference	Depth (m)	Re (ppb)	±	Os (ppt)	±	^{187}Re / ^{188}Os	±	^{187}Os / ^{188}Os	±	^{192}Os (ppt)	Os_g (@ 53 Ma)	Os_g (@ 68 Ma)	Os_g (@83 Ma)	±
AD95-10	(27)	1028.78	183.4	0.60	1663.0	5.29	627.4	2.54	1.514	0.004	581.4	0.959	0.802	0.646	0.001
AD97-10	(27)	1029.65	198.2	0.65	1817.4	5.89	627.5	2.50	1.616	0.005	628.3	1.062	0.905	0.748	0.002
AD99-10	(27)	1031.35	18.8	0.07	455.1	2.32	217.5	1.69	0.821	0.008	172.3	0.629	0.575	0.520	0.003
AD100-10	(27)	1031.56	34.2	0.13	642.2	4.03	283.1	2.72	0.926	0.011	240.1	0.676	0.605	0.534	0.004
AD101-10	(27)	1031.87	79.1	0.26	1599.1	6.46	265.5	1.37	1.004	0.006	592.4	0.769	0.703	0.637	0.002
AD102-10	(27)	1035.28	0.3	0.01	39.8	1.44	39.6	3.59	0.282	0.033	16.1	0.247	0.238	0.228	0.016
AD103-10	(27)	1035.50	1.7	0.01	200.8	1.97	42.9	0.93	0.301	0.009	81.0	0.263	0.252	0.241	0.004
AD104-10	(27)	1035.69	1.8	0.02	348.9	4.40	25.5	0.74	0.292	0.011	141.0	0.269	0.263	0.256	0.006
AD105-10	(27)	1035.89	2.5	0.01	354.7	3.42	34.1	0.72	0.260	0.007	143.9	0.230	0.221	0.212	0.004
AD106-10	(27)	1036.10	12.4	0.05	1042.3	5.34	58.5	0.59	0.282	0.004	421.8	0.230	0.216	0.201	0.002
AD108-10	(27)	1036.51	11.4	0.05	1868.2	8.10	29.8	0.27	0.226	0.003	761.5	0.200	0.192	0.185	0.001
AD109-10	(27)	1036.72	48.1	0.16	6144.1	12.50	38.2	0.17	0.216	0.001	2507.5	0.182	0.173	0.163	0.000
AD110-10	(27)	1036.97	6.6	0.04	2465.9	16.09	13.0	0.19	0.177	0.003	1011.5	0.166	0.162	0.159	0.002
AD111-10	(27)	1037.21	13.7	0.05	2195.9	11.35	30.3	0.33	0.216	0.003	896.2	0.190	0.182	0.174	0.002
AD112-10	(27)	1037.41	51.4	0.17	5239.7	12.02	48.1	0.22	0.272	0.001	2123.1	0.230	0.218	0.206	0.001
AD113-10	(27)	1037.54	104.6	0.35	4429.2	10.05	117.3	0.51	0.357	0.001	1775.3	0.254	0.225	0.195	0.001
AD114-10	(27)	1037.69	122.5	0.40	8478.9	17.56	71.2	0.30	0.305	0.001	3420.9	0.243	0.225	0.207	0.001
AD115-10	(27)	1037.89	122.9	0.40	9101.5	18.69	65.9	0.28	0.223	0.001	3711.4	0.164	0.148	0.131	0.000
AD116-10	(27)	1038.12	0.5	0.01	571.5	10.23	4.6	0.21	0.171	0.010	234.6	0.167	0.166	0.165	0.006
AD117-10	(27)	1038.35	177.5	0.58	9451.3	14.93	92.6	0.34	0.304	0.001	3814.2	0.222	0.199	0.176	0.000
AD118-10	(27)	1038.50	280.3	0.95	14395.4	24.15	96.5	0.36	0.346	0.001	5778.3	0.261	0.236	0.212	0.000
AD119-10	(27)	1038.87	356.4	1.16	12387.3	16.94	142.6	0.49	0.348	0.001	4970.8	0.222	0.187	0.151	0.000
AD120-10	(27)	1039.26	77.4	0.26	1713.0	5.29	236.1	1.08	0.772	0.003	652.3	0.564	0.505	0.445	0.001
AD121-10	(27)	1039.62	0.4	0.01	45.8	0.87	46.8	2.03	0.548	0.031	17.9	0.507	0.495	0.484	0.017
AD122-10	(27)	1039.78	13.9	0.05	394.9	2.22	185.3	1.62	0.864	0.009	148.7	0.700	0.653	0.607	0.004
AD123-10	(27)	1044.20	1.0	0.01	105.7	2.07	51.1	2.17	0.773	0.044	40.2	0.728	0.715	0.703	0.025
AD124-10	(27)	1044.43	0.9	0.01	36.6	0.58	122.2	4.31	0.774	0.033	13.9	0.666	0.636	0.605	0.016
AD125-10	(27)	1044.86	0.4	0.01	38.2	0.79	60.1	3.10	0.720	0.043	14.7	0.667	0.652	0.636	0.025
AD126-10	(27)	1045.44	0.8	0.01	88.4	1.71	46.4	2.03	0.670	0.038	34.1	0.629	0.618	0.606	0.022
AD127-10	(27)	1045.51	1.1	0.01	125.9	2.42	45.2	1.90	0.656	0.037	48.6	0.616	0.605	0.593	0.021
AD129-10	(27)	1045.83	2.3	0.01	112.3	0.96	107.1	1.69	0.781	0.016	42.7	0.686	0.659	0.633	0.008
AD131-10	(27)	1046.52	0.3	0.01	56.3	0.80	23.2	1.28	0.551	0.022	22.0	0.531	0.525	0.519	0.018
AD137-10	(27)	1048.51	2.4	0.01	111.0	0.97	112.9	1.78	0.851	0.017	41.9	0.751	0.723	0.695	0.009
AD139-10	(27)	1048.97	1.4	0.02	73.9	1.51	99.5	4.51	0.804	0.047	28.0	0.716	0.691	0.666	0.024
AD141-10	(27)	1050.59	13.2	0.05	456.3	2.16	154.5	1.02	0.973	0.007	169.7	0.837	0.798	0.759	0.004

Sample ID	Reference	Depth (m)	Re (ppb)	±	Os (ppt)	±	^{187}Re / ^{188}Os	±	^{187}Os / ^{188}Os	±	^{192}Os (ppt)	Os_g (@ 53 Ma)	Os_g (@ 68 Ma)	Os_g (@83 Ma)	±
AD143-10	(27)	1051.23	18.4	0.07	576.1	2.97	171.0	1.26	0.972	0.008	214.3	0.821	0.778	0.735	0.004
Vocontian Basin (Pont d'Issole (Adx-10 and ISLx) and Vergons (VGN) sections), SE France (Fig. 6C)															
AD41-10	(27)	17.22	0.2	0.02	306.2	1.59	3.8	0.3	1.128	0.009	111.8	1.124	1.123	1.122	0.050
AD43-10	(27)	15.55	0.1	0.02	228.8	1.47	3.1	0.4	0.937	0.012	85.4	0.934	0.934	0.933	0.066
AD45-10	(27)	15.26	13.2	0.05	324.4	1.72	217.8	1.7	0.994	0.009	120.3	0.802	0.747	0.693	0.004
AD47-10	(27)	14.86	0.1	0.02	284.4	1.93	2.1	0.4	0.943	0.013	106.1	0.941	0.940	0.940	0.080
AD49-10	(27)	14.46	0.3	0.02	6.5	0.45	227.7	41.1	0.963	0.168	2.4	0.762	0.705	0.648	0.076
AD51-10	(27)	14.06	1.2	0.02	83.8	1.62	69.0	3.1	0.427	0.025	33.3	0.366	0.349	0.331	0.012
AD55-10	(27)	13.26	0.8	0.02	109.3	3.89	37.0	3.1	0.524	0.060	42.9	0.491	0.482	0.472	0.033
AD59-10	(27)	12.46	12.5	0.05	340.7	1.71	188.4	1.6	0.629	0.006	132.0	0.463	0.415	0.368	0.002
AD61-10	(27)	12.06	1.4	0.03	149.1	5.27	47.5	3.9	0.405	0.046	59.4	0.363	0.351	0.339	0.023
AD63-10	(27)	11.66	12.8	0.05	600.9	2.56	108.5	0.8	0.558	0.005	234.9	0.463	0.436	0.408	0.002
AD64-10	(27)	11.46	14.0	0.05	461.8	2.04	154.9	1.1	0.574	0.005	180.1	0.437	0.398	0.359	0.002
AD65-10	(27)	11.26	7.5	0.03	293.4	1.85	129.8	1.5	0.520	0.008	115.2	0.406	0.373	0.341	0.003
AD67-10	(27)	10.86	10.3	0.04	366.1	2.00	143.9	1.3	0.582	0.007	142.7	0.455	0.419	0.383	0.003
AD69-10	(27)	10.46	1.5	0.01	671.1	12.09	11.1	0.5	0.220	0.012	273.8	0.210	0.207	0.205	0.007
AD71-10	(27)	10.06	2.3	0.02	733.7	13.23	15.2	0.6	0.218	0.012	299.4	0.204	0.201	0.197	0.007
AD73-10	(27)	9.66	2.4	0.01	697.4	12.52	16.8	0.7	0.189	0.011	285.6	0.174	0.170	0.166	0.006
AD75-10	(27)	9.45	0.4	0.01	1642.1	57.51	1.1	0.1	0.162	0.018	674.9	0.161	0.161	0.161	0.011
VGN 437	(27)	8.00	7.6	0.03	362.9	6.83	106.5	4.3	0.537	0.030	142.2	0.443	0.416	0.390	0.013
AD02-10	(27)	7.75	0.4	0.01	461.2	8.27	4.7	0.2	0.189	0.011	188.9	0.185	0.184	0.183	0.007
AD05-10	(27)	7.15	0.2	0.00	269.8	9.46	3.2	0.3	0.210	0.024	110.2	0.207	0.206	0.205	0.014
AD07-10	(27)	6.75	14.4	0.05	1294.0	4.82	55.3	0.4	0.361	0.003	518.4	0.312	0.298	0.284	0.001
AD09-10	(27)	6.35	4.4	0.03	670.8	6.37	31.8	0.7	0.223	0.006	273.5	0.195	0.187	0.179	0.003
AD11-10	(27)	5.95	5.9	0.02	748.4	3.22	38.2	0.3	0.215	0.002	305.5	0.181	0.171	0.162	0.001
AD13-10	(27)	5.55	4.0	0.04	651.2	2.75	29.7	0.4	0.184	0.002	266.9	0.158	0.150	0.143	0.001
AD15-10	(27)	5.15	463.9	1.50	37827.0	42.56	60.3	0.2	0.287	0.000	15297.3	0.234	0.219	0.204	0.000
AD17-10	(27)	4.75	5.1	0.02	790.9	7.30	31.2	0.6	0.175	0.005	324.5	0.147	0.139	0.132	0.002
AD19-10	(27)	4.35	11.5	0.04	2853.1	8.39	19.6	0.1	0.223	0.002	1163.4	0.206	0.201	0.196	0.001
AD21-10	(27)	3.95	5.4	0.02	1675.3	7.06	15.7	0.1	0.178	0.002	687.1	0.164	0.160	0.156	0.001
AD22-10	(27)	3.75	2.0	0.01	602.4	5.73	16.0	0.3	0.206	0.006	246.2	0.191	0.187	0.183	0.003
AD23-10	(27)	3.55	1.3	0.01	623.4	11.20	10.3	0.4	0.201	0.011	254.9	0.192	0.189	0.187	0.006
AD24-10	(27)	3.35	4.4	0.02	275.3	2.90	80.2	1.7	0.430	0.013	109.3	0.359	0.339	0.319	0.006
ISL8	(27)	2.80	18.5	0.06	4084.2	10.75	22.1	0.1	0.250	0.001	1659.5	0.231	0.225	0.220	0.001

Sample ID	Reference	Depth (m)	Re (ppb)	±	Os (ppt)	±	$^{187}Re / ^{188}Os$	±	$^{187}Os / ^{188}Os$	±	^{192}Os (ppt)	Os_g (@ 53 Ma)	Os_g (@ 68 Ma)	Os_g (@83 Ma)	±
ISL7	(27)	2.30	0.4	0.01	179.6	3.37	11.7	0.6	0.510	0.029	70.6	0.500	0.497	0.494	0.019
VGN 436.5	(27)	1.50	0.9	0.03	167.2	0.85	25.9	0.9	0.257	0.004	67.9	0.234	0.228	0.221	0.004
VGN 436	(27)	1.00	0.3	0.01	31.2	0.63	52.2	2.4	0.896	0.052	11.7	0.850	0.837	0.823	0.030
VGN 435	(27)	0.00	0.2	0.01	30.2	0.61	36.4	1.8	0.825	0.048	11.4	0.793	0.784	0.775	0.030
AD27-10	(27)	-0.78	0.4	0.01	31.1	1.12	66.0	5.5	0.896	0.102	11.7	0.838	0.821	0.805	0.056
AD29-10	(27)	-1.07	0.3	0.00	35.7	1.29	40.2	3.3	0.902	0.102	13.4	0.866	0.856	0.846	0.059
AD33-10	(27)	-1.84	0.3	0.00	57.0	1.53	28.1	1.6	0.975	0.079	21.2	0.950	0.943	0.936	0.046
VGN 431	(27)	-4.00	0.5	0.01	33.8	0.72	74.4	3.8	0.892	0.054	12.7	0.826	0.808	0.789	0.031
Wunstorf, NW Germany (Fig. 6D)															
AD44-09	(27)	-27.17	2.6	0.01	70.7	0.46	194.9	2.0	0.919	0.012	26.5	0.747	0.698	0.649	0.010
AD42-09	(27)	-28.76	1.0	0.01	53.6	0.36	95.6	1.2	0.818	0.011	20.3	0.734	0.710	0.686	0.012
AD41-09	(27)	-29.4	1.6	0.01	44.2	0.32	191.9	2.3	0.949	0.013	16.5	0.779	0.731	0.683	0.012
AD40-09	(27)	-30.06	14.9	0.05	505.8	1.43	156.3	0.6	0.907	0.003	189.5	0.769	0.730	0.691	0.003
AD38-09	(27)	-32.72	1.1	0.01	118.7	0.68	48.1	0.5	0.688	0.008	45.7	0.646	0.634	0.622	0.010
AD30-09	(27)	-36.53	38.8	0.13	398.1	0.97	551.6	1.9	1.456	0.003	140.1	0.969	0.831	0.693	0.002
AD28-09	(27)	-37.63	0.5	0.01	88.2	0.34	30.4	0.4	0.608	0.004	34.3	0.582	0.574	0.566	0.008
AD27-09	(27)	-38.34	1.7	0.01	253.9	0.81	34.4	0.2	0.596	0.003	98.8	0.566	0.557	0.549	0.004
AD26-09	(27)	-39.84	5.2	0.02	288.0	0.78	93.2	0.4	0.631	0.002	111.6	0.548	0.525	0.502	0.003
AD25-09	(27)	-40.47	9.9	0.03	270.7	0.64	192.4	0.7	0.812	0.002	102.6	0.642	0.594	0.546	0.002
AD24-09	(27)	-41.1	12.4	0.04	296.7	0.65	220.6	0.8	0.837	0.002	112.1	0.642	0.587	0.532	0.002
AD23-09	(27)	-42.05	1.2	0.01	133.1	0.69	46.1	0.5	0.490	0.006	52.5	0.449	0.438	0.426	0.007
AD21-09	(27)	-43.15	6.9	0.02	737.4	2.20	47.2	0.2	0.465	0.002	291.6	0.423	0.412	0.400	0.003
AD19-09	(27)	-43.78	16.8	0.05	485.0	1.00	177.1	0.7	0.617	0.001	188.2	0.460	0.416	0.372	0.002
AD17-09	(27)	-44.33	5.0	0.02	2247.3	4.86	10.8	0.1	0.199	0.001	919.2	0.189	0.186	0.184	0.001
AD12-09	(27)	-47.7	3.4	0.01	1375.3	5.03	12.1	0.1	0.198	0.002	562.6	0.187	0.184	0.181	0.002
AD11-09	(27)	-47.97	0.7	0.01	223.7	2.13	14.5	0.3	0.232	0.007	91.1	0.220	0.216	0.212	0.008
AD10-09	(27)	-48.1	8.5	0.03	801.8	1.58	52.1	0.2	0.267	0.001	325.1	0.221	0.208	0.195	0.001
AD9-09	(27)	-48.32	3.7	0.01	551.0	1.69	32.9	0.2	0.246	0.002	224.0	0.217	0.209	0.201	0.002
ODP207, Site 1260 (Hole 1260B), Demerara Rise (Fig. 6E)															
04W/57-58.5	(26)	-424.89	NS	NS	802.6	NS	564.59	3.72	1.4695	0.0132	NS	0.971	0.83	0.688	NS
04W/67-68.5	(26)	-424.99	NS	NS	517.8	NS	512.02	2.30	1.3816	0.0045	NS	0.929	0.80	0.673	NS
04W/73-74.5R	(26)	-425.05	NS	NS	752.0	NS	1231.05	6.34	2.54	0.0103	NS	1.453	1.14	0.837	NS

Sample ID	Reference	Depth (m)	Re (ppb)	±	Os (ppt)	±	^{187}Re / ^{188}Os	±	^{187}Os / ^{188}Os	±	^{192}Os (ppt)	Os_g (@ 53 Ma)	Os_g (@ 68 Ma)	Os_g (@83 Ma)	±
04W/81-83	(26)	-425.13	NS	NS	774.9	NS	1434.53	6.14	2.7445	0.0091	NS	1.477	1.12	0.759	NS
04W/96.5-98	(26)	-425.28	NS	NS	1073.8	NS	207.27	1.14	0.6308	0.0041	NS	0.448	0.40	0.344	NS
04W/102-103.5	(26)	-425.34	NS	NS	1145.7	NS	200.49	0.78	0.6187	0.0013	NS	0.442	0.39	0.341	NS
04W/108.5-110	(26)	-425.40	NS	NS	1792.3	NS	367.38	1.59	0.8694	0.0034	NS	0.545	0.45	0.361	NS
04W/112-114R	(26)	-425.44	NS	NS	1481.5	NS	161.1	0.68	0.5106	0.0018	NS	0.368	0.33	0.288	NS
04W/122-124	(26)	-425.54	NS	NS	1314.3	NS	169.08	0.73	0.5444	0.0018	NS	0.395	0.35	0.310	NS
05W/11-13.5	(26)	-425.93	NS	NS	2115.3	NS	208.88	0.88	0.5089	0.0020	NS	0.324	0.27	0.220	NS
05W/22-24R	(26)	-426.04	NS	NS	11696.4	NS	86.75	0.33	0.2672	0.0006	NS	0.191	0.17	0.147	NS
05W/31-34R	(26)	-426.14	NS	NS	4198.6	NS	269.62	1.06	0.5813	0.0014	NS	0.343	0.28	0.208	NS
05A/43-45	(26)	-426.25	NS	NS	3531.9	NS	113.33	0.43	0.345	0.0006	NS	0.245	0.22	0.188	NS
05A/55-57	(26)	-426.37	NS	NS	8989.6	NS	163.02	0.76	0.4331	0.0022	NS	0.289	0.25	0.208	NS
05A/59-61	(26)	-426.41	NS	NS	14122.1	NS	53.26	0.22	0.2464	0.0007	NS	0.199	0.19	0.173	NS
05A/61-63	(26)	-426.43	NS	NS	5219.1	NS	94.38	0.36	0.3272	0.0007	NS	0.244	0.22	0.197	NS
05A/64-66	(26)	-426.46	NS	NS	1230.8	NS	474.33	2.02	1.1303	0.0033	NS	0.711	0.59	0.474	NS
05A/70-72	(26)	-426.52	NS	NS	649.9	NS	855.77	4.33	1.8756	0.0097	NS	1.120	0.91	0.691	NS
05A/95-97	(26)	-426.77	NS	NS	550.4	NS	1172.7	6.24	2.4651	0.0140	NS	1.429	1.14	0.842	NS
05A/110-112	(26)	-426.92	NS	NS	468.4	NS	617.98	3.35	1.6099	0.0094	NS	1.064	0.91	0.755	NS
05A/125-127	(26)	-427.07	NS	NS	460.3	NS	643.26	3.62	1.7312	0.0111	NS	1.163	1.00	0.841	NS
05A/142-144	(26)	-427.24	NS	NS	594.8	NS	2029.38	10.29	4.1732	0.0203	NS	2.381	1.87	1.365	NS
06A/10-12	(26)	-427.42	NS	NS	481.9	NS	975.96	5.07	2.2706	0.0108	NS	1.408	1.16	0.920	NS
06A/25-27	(26)	-427.57	NS	NS	334.2	NS	670.49	5.11	1.7364	0.0187	NS	1.144	0.98	0.809	NS
06A/37-38.5	(26)	-427.69	NS	NS	107.5	NS	2023.11	29.97	4.0005	0.0615	NS	2.213	1.71	1.201	NS
35/05W/125-127	(27)	-427.07	62.9	0.20	571.5	2.16	642.3	2.8	1.744	0.006	194.9	1.176	1.02	0.855	0.002
35/05W/144-147	(27)	-427.26	146.7	0.47	473.0	2.46	2316.7	11.1	4.348	0.017	125.9	2.374	1.80	1.233	0.003
35/06W/01-03	(27)	-427.32	125.2	0.40	411.4	2.21	2281.4	11.4	4.390	0.018	109.2	2.374	1.80	1.233	0.003
35/06W/55-57	(27)	-427.87	35.5	0.12	246.2	1.49	874.2	6.3	2.104	0.016	80.8	1.331	1.11	0.894	0.004
36/01W/14-17	(27)	-428.24	33.7	0.11	112.2	1.25	2214.0	32.8	4.197	0.067	30.3	2.241	1.69	1.133	0.008
36/01W/54-56	(27)	-428.64	186.6	0.60	699.2	3.00	1925.4	7.8	3.938	0.011	192.8	2.238	1.76	1.274	0.002
36/01W/135-138	(27)	-429.45	68.6	0.22	565.3	2.50	717.6	3.4	1.873	0.009	190.1	1.239	1.06	0.880	0.002
36/02W/23-25	(27)	-429.83	111.4	0.36	538.3	2.49	1360.9	6.1	2.925	0.011	162.8	1.723	1.38	1.042	0.002
36/02W/83-85	(27)	-430.43	62.1	0.20	513.7	2.07	712.1	3.2	1.829	0.007	173.5	1.200	1.02	0.844	0.002
36/02W/105-108	(27)	-430.65	629.9	2.03	5126.8	29.87	717.9	4.3	1.757	0.014	1745.5	1.123	0.94	0.764	0.003
36/03W/10-12	(27)	-431.20	65.3	0.21	585.3	2.38	646.5	3.0	1.684	0.007	200.9	1.113	0.95	0.789	0.002
36/03W/51-53	(27)	-431.61	184.8	0.60	759.6	2.86	1635.5	6.3	3.157	0.008	224.8	1.713	1.30	0.894	0.001

Sample ID	Reference	Depth (m)	Re (ppb)	±	Os (ppt)	±	^{187}Re / ^{188}Os	±	^{187}Os / ^{188}Os	±	^{192}Os (ppt)	Os_g (@ 53 Ma)	Os_g (@ 68 Ma)	Os_g (@83 Ma)	±
Furlo, Italy (Fig. 6F)															
BON-16	(26)	-3.05	NS	NS	1451	NS	302.62	1.22	0.9341	0.0020	NS	0.515	0.591	0.667	NS
BON-14	(26)	-3.14	NS	NS	1654	NS	454.07	1.85	1.1783	0.0030	NS	0.550	0.664	0.777	NS
BON-12	(26)	-3.20	NS	NS	2344	NS	412.42	1.85	1.0822	0.0048	NS	0.512	0.615	0.718	NS
BON-10	(26)	-3.29	NS	NS	854	NS	152.21	0.73	0.6415	0.0027	NS	0.431	0.469	0.507	NS
BON-9	(26)	-3.40	NS	NS	2764	NS	169.87	0.64	0.5821	0.0010	NS	0.347	0.390	0.432	NS
BON-8	(26)	-3.48	NS	NS	2492	NS	201.51	0.80	0.603	0.0015	NS	0.324	0.375	0.425	NS
BON-6	(26)	-3.56	NS	NS	1471	NS	234.36	0.97	0.643	0.0018	NS	0.319	0.377	0.436	NS
BON-5	(26)	-3.75	NS	NS	5060	NS	156.38	0.60	0.424	0.0009	NS	0.208	0.247	0.286	NS
BON-4	(26)	-3.85	NS	NS	15002	NS	86.68	0.32	0.2922	0.0004	NS	0.172	0.194	0.216	NS
BON-3	(26)	-3.94	NS	NS	6893	NS	45.3	0.17	0.2347	0.0004	NS	0.172	0.183	0.195	NS
BON-2	(26)	-3.98	NS	NS	10691	NS	59.12	0.22	0.2452	0.0004	NS	0.163	0.178	0.193	NS
BON-1	(26)	-4.03	NS	NS	10385	NS	77.59	0.32	0.2716	0.0008	NS	See Furlo 3.6	See Furlo 3.6	See Furlo 3.6	NS
BON-A1	(26)	-4.45	NS	NS	114	NS	64.6	0.67	0.7567	0.0079	NS	0.667	0.683	0.700	NS
BON-B1	(26)	-9.17	NS	NS	117	NS	116.33	2.21	0.8864	0.0214	NS	0.725	0.755	0.784	NS
BON-C1	(26)	-13.15	NS	NS	126	NS	85.72	0.84	0.9824	0.0097	NS	0.864	0.885	0.907	NS
BON-D1	(26)	-16.06	NS	NS	141	NS	185.61	2.80	1.2059	0.0183	NS	0.949	0.996	1.042	NS
BON-E1	(26)	-20.54	NS	NS	138	NS	131.03	1.16	1.0436	0.0093	NS	0.862	0.895	0.928	NS
Furlo 8.1	(27)	-3.99	81.1	0.27	10316.0	17.40	38.4	0.2	0.235	0.001	0.4	0.182	0.192	0.201	0.000
Furlo 3.6	(27)	-4.03	130.4	0.43	11355.4	16.46	56.4	0.2	0.272	0.001	0.3	0.194	0.208	0.222	0.000
Furlo 0	(27)	-4.07	0.3	0.01	163.8	2.06	8.1	0.3	0.291	0.011	0.5	0.279	0.281	0.283	0.007
Furlo -42	(27)	-4.49	3.4	0.02	55.3	0.51	330.0	5.4	1.059	0.020	0.8	0.603	0.685	0.768	0.007
Furlo -70	(27)	-4.77	1.0	0.01	42.1	0.64	126.9	4.2	0.743	0.031	0.7	0.568	0.600	0.631	0.014
Furlo -90	(27)	-4.97	1.5	0.01	57.2	0.69	134.7	3.3	0.754	0.023	0.7	0.568	0.602	0.635	0.011
Yezo Group, Japan (Fig. 6G)															
T740A	(28)	138.10	0.30	0.01	39.27	0.55	39.6	1.4	0.669	0.026	15.14	0.63	0.62	0.61	0.02
T738A	(28)	136.53	0.34	0.01	45.96	0.51	38.2	1.1	0.669	0.020	17.72	0.63	0.63	0.62	0.01
T736A	(28)	133.96	0.34	0.01	48.05	0.66	36.0	1.2	0.654	0.025	18.56	0.62	0.61	0.60	0.02
T733A	(28)	130.55	0.33	0.01	51.12	0.70	33.4	1.1	0.653	0.025	19.75	0.62	0.62	0.61	0.02
T730A	(28)	127.25	0.42	0.01	41.83	0.47	52.0	1.3	0.671	0.020	16.13	0.63	0.61	0.60	0.01
T728A	(28)	125.40	0.35	0.01	52.53	0.73	34.4	1.1	0.683	0.026	20.22	0.65	0.64	0.64	0.02
T726A	(28)	123.60	0.32	0.01	46.17	0.64	36.1	1.2	0.656	0.025	17.83	0.62	0.62	0.61	0.02
T724A	(28)	121.20	0.43	0.01	41.02	0.46	54.3	1.4	0.684	0.020	15.79	0.64	0.62	0.61	0.01
T719A	(28)	116.00	0.40	0.01	47.67	0.66	42.9	1.4	0.686	0.026	18.34	0.65	0.64	0.63	0.02

Sample ID	Reference	Depth (m)	Re (ppb)	±	Os (ppt)	±	^{187}Re / ^{188}Os	±	^{187}Os / ^{188}Os	±	^{192}Os (ppt)	Os_g (@ 53 Ma)	Os_g (@ 68 Ma)	Os_g (@83 Ma)	±
T717A	(28)	113.96	0.25	0.01	37.26	0.73	35.0	1.7	0.658	0.038	14.39	0.63	0.62	0.61	0.02
T715A	(28)	111.75	0.29	0.01	38.82	0.55	38.2	1.3	0.673	0.026	14.96	0.64	0.63	0.62	0.02
T713A	(28)	110.00	0.32	0.01	41.11	0.57	40.4	1.4	0.655	0.026	15.88	0.62	0.61	0.60	0.02
T710A	(28)	104.36	0.30	0.01	35.53	0.50	43.5	1.5	0.679	0.027	13.68	0.64	0.63	0.62	0.02
AD90-11	(28)	98.75	0.23	0.01	27.86	0.55	42.0	2.1	0.695	0.040	10.71	0.66	0.65	0.64	0.02
AD92-11	(28)	92.50	0.32	0.01	47.22	0.92	34.7	1.6	0.684	0.039	18.17	0.65	0.64	0.64	0.02
AD94-11	(28)	84.10	0.28	0.01	41.37	0.81	35.2	1.6	0.690	0.040	15.91	0.66	0.65	0.64	0.02
AD96-11	(28)	77.10	0.45	0.01	42.20	0.82	54.8	2.4	0.629	0.036	16.35	0.58	0.57	0.55	0.02
AD98-11	(28)	71.85	0.23	0.01	33.33	0.65	35.4	1.7	0.662	0.038	12.86	0.63	0.62	0.61	0.02
AD100-11	(28)	64.85	0.25	0.01	42.66	0.83	30.2	1.4	0.678	0.039	16.43	0.65	0.64	0.64	0.02
AD102-11	(28)	59.25	0.29	0.01	40.54	0.79	37.5	1.7	0.663	0.038	15.64	0.63	0.62	0.61	0.02
AD104-11	(28)	50.75	0.28	0.01	40.28	0.78	35.1	1.6	0.600	0.035	15.67	0.57	0.56	0.55	0.02
AD106-11	(28)	44.00	0.16	0.01	29.15	0.58	27.4	1.6	0.640	0.038	11.28	0.62	0.61	0.60	0.02
AD108-11	(28)	36.90	0.17	0.01	34.34	0.67	25.2	1.4	0.646	0.037	13.28	0.62	0.62	0.61	0.02
AD110-11	(28)	29.50	0.32	0.01	40.30	0.78	40.4	1.8	0.645	0.037	15.58	0.61	0.60	0.59	0.02
AD112-11	(28)	22.75	0.20	0.01	28.27	0.56	36.2	1.9	0.629	0.037	10.95	0.60	0.59	0.58	0.02
AD114-11	(28)	15.80	0.16	0.01	30.29	0.59	27.7	1.5	0.605	0.035	11.77	0.58	0.57	0.57	0.02
AD116-11	(28)	7.90	0.26	0.01	32.99	0.64	39.6	1.9	0.563	0.033	12.89	0.53	0.52	0.51	0.02
AD118-11	(28)	0.25	0.32	0.01	35.19	0.68	46.4	2.1	0.614	0.035	13.66	0.57	0.56	0.55	0.02
AD120-11	(28)	-7.15	1.10	0.01	307.60	5.54	17.4	0.7	0.205	0.012	125.71	0.19	0.19	0.18	0.01
AD121-11	(28)	-11.25	1.17	0.01	580.82	10.41	9.8	0.4	0.179	0.010	238.18	0.17	0.17	0.17	0.01
AD122-11	(28)	-15.20	1.40	0.01	738.64	13.25	9.2	0.4	0.187	0.011	302.60	0.18	0.18	0.17	0.01
CT056	(28)	-16.15	1.43	0.01	236.71	2.24	29.3	0.6	0.195	0.006	96.87	0.17	0.16	0.15	0.00
CT055	(28)	-17.40	0.73	0.01	11.45	0.35	331.1	23.5	0.708	0.059	4.39	0.42	0.33	0.25	0.01
CT053	(28)	-18.60	0.55	0.01	8.98	0.32	322.5	27.4	0.787	0.076	3.41	0.50	0.42	0.34	0.02
AD123-11	(28)	-19.35	1.24	0.01	61.92	1.25	105.8	4.5	0.860	0.050	23.33	0.77	0.74	0.71	0.03
AD124-11	(28)	-21.00	1.19	0.01	57.71	1.17	109.0	4.7	0.875	0.051	21.71	0.78	0.75	0.72	0.03
AD125-11	(28)	-23.25	1.01	0.01	39.24	0.83	136.3	6.2	0.914	0.055	14.69	0.79	0.76	0.73	0.03
AD126-11	(28)	-24.95	1.04	0.01	60.22	1.19	90.9	3.8	0.838	0.048	22.75	0.76	0.73	0.71	0.02
AD128-11	(28)	-28.55	1.50	0.01	63.65	1.29	125.5	5.3	0.920	0.053	23.82	0.81	0.78	0.75	0.03
AD130-11	(28)	-33.70	1.04	0.01	58.92	1.18	93.1	4.0	0.819	0.048	22.31	0.74	0.71	0.69	0.02
AD132-11	(28)	-37.10	0.92	0.01	58.15	1.15	83.2	3.5	0.840	0.048	21.96	0.77	0.75	0.72	0.03
AD134-11	(28)	-40.65	1.10	0.01	64.79	1.30	89.2	3.8	0.845	0.049	24.46	0.77	0.74	0.72	0.03
AD136-11	(28)	-44.60	1.21	0.01	49.14	1.01	130.1	5.7	0.876	0.051	18.48	0.76	0.73	0.70	0.02
AD138-11	(28)	-47.95	0.90	0.01	56.73	1.13	82.9	3.6	0.816	0.047	21.49	0.74	0.72	0.70	0.02

Sample ID	Reference	Depth (m)	Re (ppb)	±	Os (ppt)	±	^{187}Re / ^{188}Os	±	^{187}Os / ^{188}Os	±	^{192}Os (ppt)	Os_g (@ 53 Ma)	Os_g (@ 68 Ma)	Os_g (@83 Ma)	±
AD140-11	(28)	-51.25	1.63	0.02	70.38	1.42	122.4	5.3	0.850	0.049	26.55	0.74	0.71	0.68	0.02
AD142-11	(28)	-56.10	0.83	0.01	65.26	1.29	65.8	2.9	0.737	0.043	24.96	0.68	0.66	0.65	0.02
AD144-11	(28)	-59.85	1.10	0.01	87.96	1.23	64.8	1.9	0.686	0.027	33.85	0.63	0.61	0.60	0.01
AD146-11	(28)	-63.80	1.08	0.01	101.45	1.39	54.6	1.6	0.603	0.023	39.44	0.56	0.54	0.53	0.01
AD148-11	(28)	-67.00	0.72	0.01	50.78	1.03	74.5	3.4	0.835	0.049	19.19	0.77	0.75	0.73	0.03
AD150-11	(28)	-71.25	0.80	0.01	50.47	1.03	83.6	3.8	0.868	0.051	19.00	0.79	0.77	0.75	0.03
AD152-11	(28)	-75.00	0.89	0.01	64.57	0.60	72.1	1.6	0.787	0.017	24.55	0.72	0.71	0.69	0.01
AD154-11	(28)	-79.75	0.55	0.01	40.34	0.84	71.6	3.5	0.834	0.050	15.25	0.77	0.75	0.73	0.03
AD156-11	(28)	-83.45	1.15	0.01	64.68	0.96	94.5	2.9	0.891	0.035	24.28	0.81	0.78	0.76	0.02
AD158-11	(28)	-88.00	0.82	0.01	45.48	0.93	94.3	4.3	0.828	0.049	17.20	0.74	0.72	0.70	0.03
AD160-11	(28)	-91.20	0.58	0.01	47.51	0.97	64.3	3.1	0.832	0.049	17.96	0.78	0.76	0.74	0.03
AD162-11	(28)	-94.40	0.59	0.01	44.56	0.92	70.8	3.4	0.913	0.054	16.68	0.85	0.83	0.82	0.03
AD164-11	(28)	-97.70	0.93	0.01	47.92	0.98	101.7	4.5	0.814	0.048	18.16	0.72	0.70	0.67	0.02
AD166-11	(28)	-101.60	0.72	0.01	46.59	0.96	81.8	3.8	0.845	0.050	17.59	0.77	0.75	0.73	0.03
AD168-11	(28)	-105.10	0.67	0.01	42.54	0.88	83.3	3.9	0.823	0.049	16.10	0.75	0.73	0.71	0.03
AD170-11	(28)	-109.60	0.75	0.01	46.35	0.94	85.1	3.9	0.796	0.047	17.60	0.72	0.70	0.68	0.02
AD172-11	(28)	-112.70	0.83	0.01	57.31	1.16	76.4	3.4	0.864	0.050	21.58	0.80	0.78	0.76	0.03
AD174-11	(28)	-116.10	0.67	0.01	40.82	0.85	85.6	4.0	0.810	0.048	15.48	0.73	0.71	0.69	0.03
AD176-11	(28)	-120.30	0.76	0.01	44.90	0.92	89.7	4.1	0.840	0.050	16.96	0.76	0.74	0.72	0.03
AD178-11	(28)	-124.30	0.76	0.01	54.98	1.12	72.6	3.3	0.837	0.049	20.77	0.77	0.75	0.74	0.03
AD180-11	(28)	-131.70	0.33	0.01	30.16	1.13	57.7	5.3	0.801	0.094	11.45	0.75	0.74	0.72	0.05
**Great Valley Sequence, California, USA (Fig. 6H)**															
US 908	(28)	312.50	1.03	0.01	85.03	1.68	63.5	2.7	0.818	0.047	32.20	0.76	0.75	0.73	0.03
US 903	(28)	296.00	0.76	0.01	65.38	1.30	61.0	2.7	0.765	0.044	24.92	0.71	0.70	0.68	0.02
US 089	(28)	279.50	0.87	0.01	74.35	1.47	60.9	2.6	0.765	0.046	28.34	0.71	0.70	0.68	0.02
US 613	(28)	261.00	0.32	0.01	24.10	0.38	71.9	2.6	1.008	0.041	8.92	0.94	0.93	0.91	0.02
US 621	(28)	244.50	0.48	0.01	53.99	0.75	45.9	1.4	0.681	0.026	20.79	0.64	0.63	0.62	0.01
US 626	(28)	227.50	0.38	0.01	40.95	0.81	48.7	2.1	0.764	0.044	15.61	0.72	0.71	0.70	0.02
US 632	(28)	212.80	0.45	0.01	49.77	0.70	46.6	1.4	0.746	0.029	19.01	0.71	0.69	0.68	0.02
US 637	(28)	195.50	0.61	0.01	56.59	1.13	56.4	2.6	0.730	0.043	21.66	0.68	0.67	0.65	0.02
US 648	(28)	175.20	0.29	0.01	54.13	1.03	27.7	1.3	0.588	0.034	21.08	0.56	0.56	0.55	0.02
US 653	(28)	159.50	1.05	0.01	82.73	1.60	65.2	2.8	0.606	0.035	32.15	0.55	0.53	0.52	0.02
US 084	(28)	142.00	0.89	0.01	140.46	2.65	32.0	1.4	0.462	0.027	55.56	0.43	0.43	0.42	0.01
US 662	(28)	124.50	0.48	0.01	156.16	1.98	15.1	0.4	0.333	0.013	62.78	0.32	0.32	0.31	0.01

Sample ID	Reference	Depth (m)	Re (ppb)	±	Os (ppt)	±	^{187}Re / ^{188}Os	±	^{187}Os / ^{188}Os	±	^{192}Os (ppt)	Os_g (@ 53 Ma)	Os_g (@ 68 Ma)	Os_g (@83 Ma)	±
US 666	(28)	106.20	0.62	0.01	179.10	3.26	16.9	0.8	0.262	0.015	72.66	0.25	0.24	0.24	0.01
US 674	(28)	88.50	1.05	0.01	154.02	2.85	34.0	1.4	0.388	0.022	61.50	0.36	0.35	0.34	0.01
US 675	(28)	84.10	0.86	0.01	223.71	4.09	18.9	0.8	0.298	0.017	90.35	0.28	0.28	0.27	0.01
US 061	(28)	82.00	0.92	0.01	176.47	3.25	25.9	1.1	0.346	0.020	70.84	0.32	0.32	0.31	0.01
US 676	(28)	78.90	0.80	0.01	32.30	0.65	122.7	5.8	0.309	0.020	13.03	0.20	0.17	0.14	0.00
US 677	(28)	75.60	0.81	0.01	30.52	0.62	130.2	6.2	0.311	0.020	12.30	0.20	0.16	0.13	0.00
US 062	(28)	72.30	1.28	0.01	424.53	5.26	14.7	0.4	0.239	0.009	172.75	0.23	0.22	0.22	0.01
US 678	(28)	68.50	1.58	0.01	108.22	1.08	71.7	1.6	0.259	0.008	43.92	0.20	0.18	0.16	0.00
US 679	(28)	65.00	0.59	0.01	67.24	1.25	43.1	2.0	0.218	0.013	27.43	0.18	0.17	0.16	0.01
US 680	(28)	60.70	1.34	0.01	88.28	0.91	75.0	1.7	0.305	0.009	35.62	0.24	0.22	0.20	0.00
US 681	(28)	60.40	0.92	0.01	39.94	0.78	113.3	5.1	0.317	0.019	16.09	0.22	0.19	0.16	0.01
US 682	(28)	59.90	1.94	0.01	36.54	0.48	270.3	7.4	0.558	0.019	14.28	0.32	0.25	0.18	0.00
US 684	(28)	58.90	1.31	0.01	121.25	1.37	56.7	1.3	0.790	0.023	46.07	0.74	0.73	0.71	0.01
US 063	(28)	58.00	1.49	0.01	28.23	0.42	274.9	8.7	0.752	0.028	10.78	0.51	0.44	0.37	0.01
US 687	(28)	57.00	1.24	0.01	20.34	0.37	322.1	12.6	0.889	0.039	7.64	0.60	0.52	0.44	0.01
US 688	(28)	55.71	1.30	0.01	28.38	0.42	237.8	7.5	0.726	0.027	10.87	0.52	0.46	0.40	0.01
US 064	(28)	55.60	1.07	0.01	14.23	0.39	396.3	24.7	0.840	0.063	5.38	0.49	0.39	0.29	0.01
US 065	(28)	55.30	2.34	0.01	30.70	0.40	397.1	10.7	0.761	0.024	11.71	0.41	0.31	0.21	0.00
US 066	(28)	54.20	0.98	0.01	40.23	0.43	126.4	3.1	0.686	0.018	15.48	0.57	0.54	0.51	0.01
US 690	(28)	52.70	0.56	0.01	12.30	0.36	240.6	16.8	0.827	0.066	4.65	0.61	0.55	0.49	0.02
US 067	(28)	52.30	0.97	0.01	9.93	0.32	534.7	39.4	1.134	0.090	3.62	0.66	0.53	0.39	0.02
US 691	(28)	51.60	0.75	0.01	17.71	0.44	218.9	12.4	0.680	0.048	6.82	0.49	0.43	0.38	0.02
US 692	(28)	50.40	0.99	0.01	92.01	1.31	56.4	1.7	0.810	0.031	34.88	0.76	0.75	0.73	0.02
US 068	(28)	50.20	0.67	0.01	17.58	0.44	199.5	11.5	0.752	0.052	6.71	0.58	0.53	0.48	0.02
US 069	(28)	49.50	1.17	0.01	13.93	0.34	443.1	24.2	0.827	0.052	5.27	0.44	0.32	0.21	0.01
US 694	(28)	48.10	1.10	0.01	12.77	0.33	457.2	26.9	0.875	0.058	4.80	0.47	0.36	0.24	0.01
US 070	(28)	47.70	1.22	0.01	21.53	0.40	290.3	12.0	0.640	0.032	8.33	0.38	0.31	0.24	0.01
US 071	(28)	45.20	0.57	0.01	17.82	0.43	160.8	9.3	0.530	0.037	6.99	0.39	0.35	0.31	0.01
US 072	(28)	44.50	3.74	0.02	61.54	0.62	322.0	5.7	0.898	0.022	23.08	0.61	0.53	0.45	0.01
US 698	(28)	43.90	2.21	0.01	36.81	0.42	316.1	7.2	0.827	0.022	13.93	0.55	0.47	0.39	0.01
US 1002	(28)	43.20	0.69	0.01	15.18	0.40	235.8	14.5	0.744	0.055	5.80	0.54	0.48	0.42	0.02
US 1005	(28)	40.40	1.45	0.01	31.97	0.45	236.6	7.0	0.751	0.027	12.21	0.54	0.48	0.42	0.01
US 1007	(28)	34.50	0.63	0.01	32.40	0.72	106.6	5.3	1.119	0.068	11.85	1.03	1.00	0.97	0.04
US 1009	(28)	27.00	1.57	0.01	22.33	0.39	377.4	13.8	0.988	0.042	8.29	0.65	0.56	0.47	0.01
US 075	(28)	25.50	1.12	0.01	36.12	0.61	170.1	5.9	1.188	0.051	13.10	1.04	1.00	0.95	0.03

Sample ID	Reference	Depth (m)	Re (ppb)	±	Os (ppt)	±	^{187}Re / ^{188}Os	±	^{187}Os / ^{188}Os	±	^{192}Os (ppt)	Os_g (@ 53 Ma)	Os_g (@ 68 Ma)	Os_g (@83 Ma)	±
US 1011	(28)	22.10	1.11	0.01	14.97	0.35	396.4	20.8	0.936	0.056	5.59	0.59	0.49	0.39	0.01
US 1012	(28)	19.40	1.35	0.01	72.41	1.46	99.6	4.2	0.949	0.055	27.00	0.86	0.84	0.81	0.03
US 076	(28)	11.00	0.82	0.01	20.56	0.40	208.7	9.2	0.794	0.040	7.81	0.61	0.56	0.51	0.02
US 077	(28)	0.00	2.74	0.01	78.35	0.61	192.2	2.5	1.212	0.018	28.34	1.04	0.99	0.95	0.01

NS = Data not specified in associated reference.
